# Circulating *N*-formylmethionine and metabolic shift in critical illness: a multicohort metabolomics study

**DOI:** 10.1186/s13054-022-04174-y

**Published:** 2022-10-19

**Authors:** Martin Ingi Sigurdsson, Hirotada Kobayashi, Karin Amrein, Kiichi Nakahira, Angela J. Rogers, Mayra Pinilla-Vera, Rebecca M. Baron, Laura E. Fredenburgh, Jessica A. Lasky-Su, Kenneth B. Christopher

**Affiliations:** 1 Anesthesiology and Critical Care Medicine, Landspitali University Hospital, University of Iceland, Hringbraut 101, 101 Reykjavík, Iceland; 2 Faculty of Medicine, University of Iceland, Vatnsmyrarvegur 16, 101 Reykjavik, Iceland; 3 Division of Renal Medicine, Brigham and Women’s Hospital, 75 Francis Street, Boston, 02115 USA; 4 Division of Endocrinology and Diabetology, Medical University of Graz, Auenbruggerplatz 15, 8036 Graz, Austria; 5 Nara Medical University, 840 Shijocho, Kashihara, Nara 634-8521 Japan; 6 Weill Cornell Medicine, 1300 York Avenue, New York, 10065 USA; 7 Stanford University Medical Center, 300 Pasteur Dr. H3143, Stanford, 94305 USA; 8 Division of Pulmonary and Critical Care Medicine, Brigham and Women’s Hospital, 75 Francis Street, Boston, 02115 USA; 9 Channing Division of Network Medicine, Brigham and Women’s Hospital, 181 Longwood Avenue, Boston, 02115 USA

**Keywords:** Metabolomics, *N*-formylmethionine, Critical illness, Acylcarnitine, Metabolic shift, Pentose phosphate pathway, Branched chain amino acids

## Abstract

**Background:**

Cell stress promotes degradation of mitochondria which release danger-associated molecular patterns that are catabolized to *N*-formylmethionine. We hypothesized that in critically ill adults, the response to *N*-formylmethionine is associated with increases in metabolomic shift-related metabolites and increases in 28-day mortality.

**Methods:**

We performed metabolomics analyses on plasma from the 428-subject Correction of Vitamin D Deficiency in Critically Ill Patients trial (VITdAL-ICU) cohort and the 90-subject Brigham and Women’s Hospital Registry of Critical Illness (RoCI) cohort. In the VITdAL-ICU cohort, we analyzed 983 metabolites at Intensive Care Unit (ICU) admission, day 3, and 7. In the RoCI cohort, we analyzed 411 metabolites at ICU admission. The association between *N*-formylmethionine and mortality was determined by adjusted logistic regression. The relationship between individual metabolites and *N*-formylmethionine abundance was assessed with false discovery rate correction via linear regression, linear mixed-effects, and Gaussian graphical models.

**Results:**

Patients with the top quartile of *N*-formylmethionine abundance at ICU admission had a significantly higher adjusted odds of 28-day mortality in the VITdAL-ICU (OR, 2.4; 95%CI 1.5–4.0; *P* = 0.001) and RoCI cohorts (OR, 5.1; 95%CI 1.4–18.7; *P* = 0.015). Adjusted linear regression shows that with increases in *N*-formylmethionine abundance at ICU admission, 55 metabolites have significant differences common to both the VITdAL-ICU and RoCI cohorts. With increased *N*-formylmethionine abundance, both cohorts had elevations in individual short-chain acylcarnitine, branched chain amino acid, kynurenine pathway, and pentose phosphate pathway metabolites.

**Conclusions:**

The results indicate that circulating *N*-formylmethionine promotes a metabolic shift with heightened mortality that involves incomplete mitochondrial fatty acid oxidation, increased branched chain amino acid metabolism, and activation of the pentose phosphate pathway.

**Graphic Abstract:**

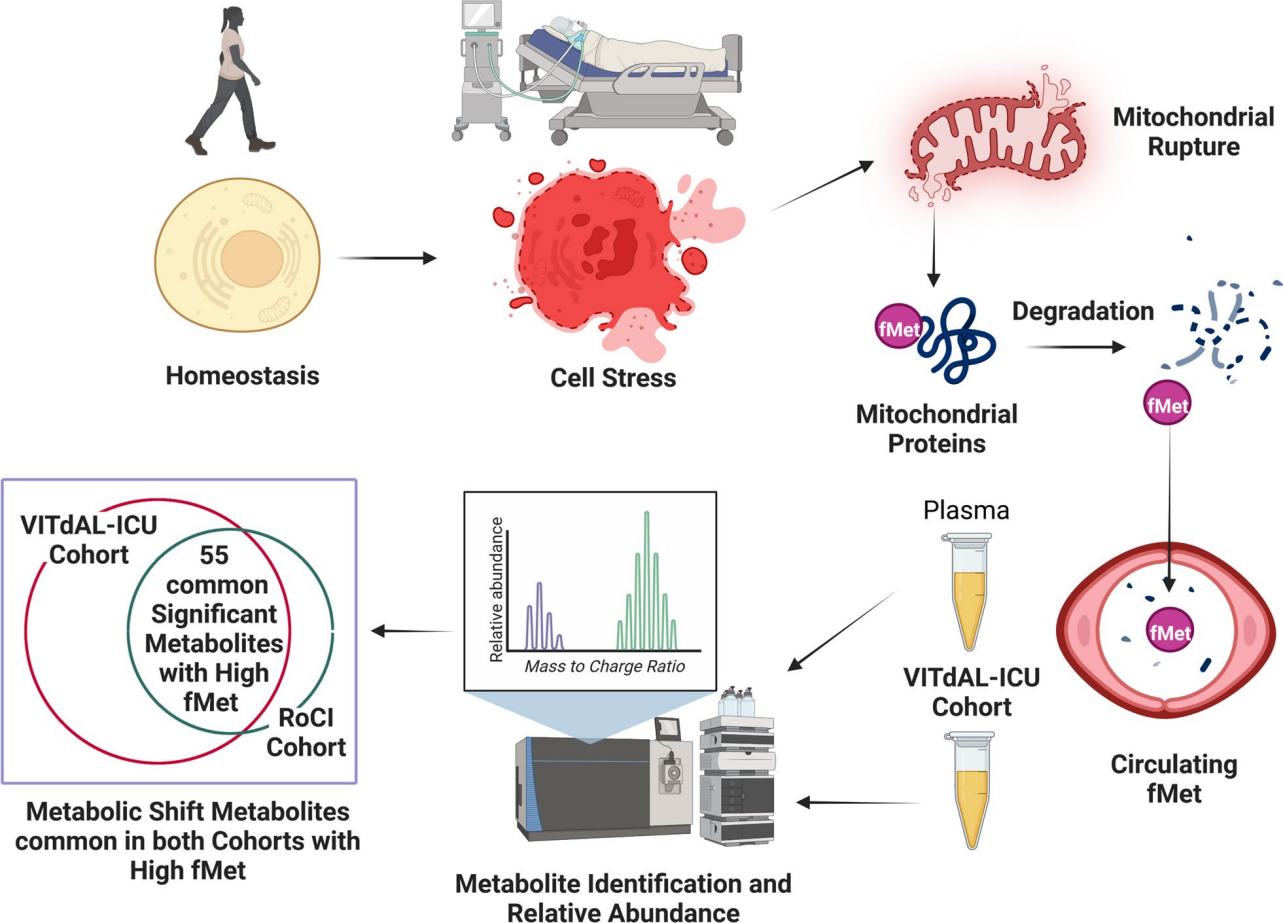

**Supplementary Information:**

The online version contains supplementary material available at 10.1186/s13054-022-04174-y.

## Background

The study of the underlying biological processes of critical illness is hindered by patient heterogeneity [1]. Defining critical illness based on common molecular mechanisms is useful for risk prediction and intervention response and can illuminate shared etiological and pathogenic pathways [2–4]. Common molecular mechanisms are described in specific populations of the critically ill using transcriptomics of COVID-19 [5], muscle transcriptomics and metabolomics in multiorgan failure [6], and genomics of sepsis due to pneumonia [7]. But these observations are not generalizable beyond the specific population studied. We argue that the molecular response to evolutionarily conserved danger stimuli may reflect common pathological mechanisms in heterogeneous critically ill patients [8].

Danger-associated molecular patterns (DAMPs) are defense signals that communicate to the whole organism that individual cells have failed to adapt to stressors [9]. In humans, degrading mitochondria yield DAMPs in the form of mitochondrial DNA and *N*-formyl-methionyl peptides. We and others have found that mitochondrial DAMPs are associated with significant dysregulation of metabolic homeostasis, potent activation of innate immunity, and adverse outcomes in the critically ill [10–12]. The further breakdown of the DAMP N-formyl-methionyl peptide (fMet-Leu-Phy) produces circulating *N*-formylmethionine that signals the substantial disruption of homeostasis [13].

Plasma metabolic profiles in the critically ill provide a way to measure point-in-time changes that reflect patients’ genetics as well as the cellular response to physiological stress, inflammation, and substrate abundance [14]. But these metabolic profiles are not routinely used to provide common molecular insights into the response to critical illness. Therefore, we studied circulating *N*-formylmethionine abundance during early critical illness to identify patients with a metabolic shift who are at the greatest risk for poor outcomes. To test the hypothesis that common differences related to plasma metabolomic shift exist in the response to *N*-formylmethionine, we performed metabolomics cohort study on plasma samples from two distinct critically ill biorepositories with clinical heterogeneity.

## Methods

### Study design and patients

The Graz, Austria-based VITdAL-ICU trial randomized 492 critically ill adults with 25-hydroxyvitamin D (25(OH)D) ≤ 20 ng/ml to high-dose oral vitamin D_3_ or placebo. Patients were enrolled from medical, neurological, cardiac surgical, and anesthesia ICUs between 2010 and 2012 and followed for 180 days [15]. Further details of the trial, consent, inclusion, and exclusion criteria are published and described in Additional file 1: Methods [15]. Plasma was obtained at days 0 (prior to randomization), 3, and 7. The 428 VITdAL-ICU trial subjects with plasma samples available comprised the VITdAL-ICU cohort [3, 16].

The Boston, USA-based Registry of Critical Illness (RoCI) is a registry and biorepository of adult medical ICU patients with SIRS, sepsis, and sepsis ARDS at the Brigham and Women’s Hospital. Details of the cohort, patient recruitment, and consent are published [17, 18] and described in e-Appendix 1. Between 2008 and 2010, 90 medical ICU patients had plasma collected within 72 h of ICU admission (defined as day 0) and these patients comprise the RoCI cohort [18].

### Metabolomics and study design

Metabolomics methods and analyses have been previously published [3, 16, 18] and are described in Additional file 1: Methods. In the VITdAL-ICU cohort, profiling for 983 metabolites in 1215 plasma samples from 428 subjects collected during the VITdAL-ICU trial was performed in 2017 [3, 15]. In the RoCI cohort, 411 metabolites were determined in 2011 on 90 patients [18]. Metabolomics data from each cohort separately underwent cube root transformation and Pareto scaling to generate data on the same scale and to approximate normal distribution.

The exposure of interest was the relative abundance of *N*-formylmethionine determined at day 0 by liquid chromatography coupled mass spectrometry by Metabolon, Inc., and analyzed as a continuous variable. We chose to study *N*-formylmethionine as it is a known catabolic product of the danger-associated molecular pattern N-formyl-methionyl peptide and was measured in both the VITdAL-ICU cohort and RoCI cohort analyzed by Metabolon, Inc. Abundance is defined as the total ion count for the given mass-to-charge ratio assigned to *N*-formylmethionine. *N*-formylmethionine was also analyzed as quartiles of abundance with elevated *N*-formylmethionine defined as abundance in the top quartile. The primary outcome was all-cause 28-day mortality determined by hospital records and patient follow-up in the VITdAL-ICU cohort and hospital records and the US Social Security Administration Death Master File in the RoCI cohort. Distributions of and differences in crude survival between quartiles of *N*-formylmethionine were determined via the Kaplan–Meier survival curve and the log-rank test, respectively. The association between quartiles of *N*-formylmethionine and 28-day mortality was determined by logistic regression adjusted for age, sex, SAPS II, admission diagnosis, and baseline 25(OH)D in the VITdAL-ICU cohort and logistic regression adjusted for age, sex, race, and APACHE II score in the RoCI cohort.

For day 0 data in the VITdAL-ICU and RoCI cohorts, Student’s t test was performed to determine if significant differences exist with elevated *N*-formylmethionine (highest quartile) using MetaboAnalyst [19]. We corrected for multiple testing via the Benjamini–Hochberg procedure to adjust the false discovery rate (FDR) to 0.05, producing a q value which was used to identify all significant differences [20]. Day 0 data were also analyzed using orthogonal partial least square-discriminant analysis (OPLS-DA) to assess the significance of classification discrimination (SIMCA 15.0 Umetrics, Sweden) [21]. OPLS-DA is a powerful statistical modeling tool that provides insights into metabolite differences between patients with and without elevated *N*-formylmethionine [22].

For single time point data in the VITdAL-ICU and RoCI cohorts, correlations between individual metabolites and *N*-formylmethionine abundance (continuous) at day 0 were determined utilizing linear regression models utilizing robust standard errors. For the VITdAL-ICU cohort, the linear regression model was corrected for age, sex, baseline 25(OH)D, SAPS II, and admission diagnosis. For the RoCI cohort, the linear regression model was adjusted for age, sex, race, and APACHE II. We performed linear regression diagnostics to evaluate model assumptions including variance inflation factor, Breusch–Pagan/Cook–Weisberg test, normality of residuals, and Cook's distance.

In the VITdAL-ICU cohort, correlations between *N*-formylmethionine and individual metabolites over days 0, 3, and 7 were also determined utilizing linear mixed-effects models correcting for age, sex, baseline 25(OH)D, absolute increase in 25(OH)D at day 3, SAPS II, plasma day, admission diagnosis, and an individual subject-specific random intercept. All linear regression and mixed-effects models were analyzed using STATA 16.1MP [23]. A *q *value threshold of 0.05 was used to identify all significant associations [20]. Rain plots were produced in R-3.6.2 [24].

Networks are useful in illustrating relationships in biological and pathophysiological pathways [25]. To identify *N*-formylmethionine-specific metabolite connections, we estimated Gaussian graphical models (GGMs) for day 0 in the VITdAL-ICU cohort [26]. Gaussian graphical models were used as they can demonstrate metabolic reactions and functional dependence between metabolites following adjustment for confounding factors [26]. We inferred a *N*-formylmethionine-specific network for relative metabolite abundance including age, sex, SAPS II, admission diagnosis, and baseline 25(OH)D as covariates into the model [27]. Edges between metabolites were allotted if both their Pearson correlations and partial correlations remained statistically significant at a q value threshold of 0.05 [20]. Gaussian graphical models were produced using the GeneNet R package, version 1.2.13 in R-3.6.2 [27].

## Results

### VITdAL-ICU cohort

**Fig. 1:**
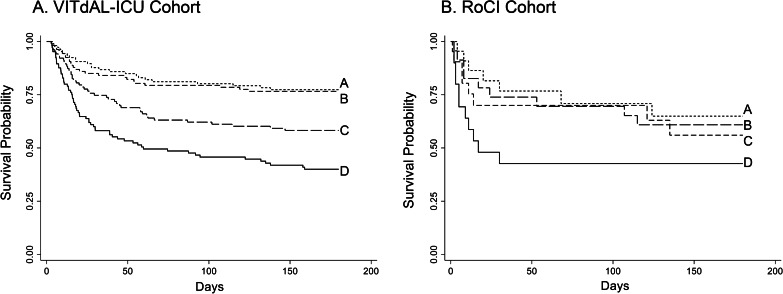
Kaplan–Meier estimates of survival. Kaplan–Meier estimates of overall survival by quartiles of plasma *N*-formylmethionine abundance at day 0 in the VITdAL-ICU (*N* = 428) Panel A and RoCI cohorts (*N* = 90) Panel B. Log-rank test revealed a significant difference in survival between the quartiles in both cohorts (*P* < 0.001 in the VITdAL-ICU cohort, *P* = 0.0476 in the RoCI cohort). **A** First quartile (lowest *N*-formylmethionine); **B** second quartile; **C** third quartile; and **D** fourth quartile (highest *N*-formylmethionine)

The baseline characteristics of the 428 subject VITdAL-ICU cohort are published and summarized in Table 1 [3, 16]. Baseline characteristics differed between subjects stratified by *N*-formylmethionine quartiles for age, sepsis, creatinine at day 0, and ICU type (Table 2; Additional file 2). The overall 28-day mortality of the 428 subject VITdAL-ICU cohort was 22%. Significant differences exist in survival between quartiles of *N*-formylmethionine abundance at day 0 (log-rank *P* < 0.001, Fig. 1A). Patients with the top quartile of plasma *N*-formylmethionine abundance at day 0 had a significantly higher odds of 28-day mortality (OR = 2.4 (95%CI 1.5–4.0; *P* = 0.001)) compared with patients in the other three quartiles, after adjustment for age, sex, 25(OH)D level, SAPS II, and admission diagnosis. Further adjustment for bilirubin, creatinine, or propofol did not materially alter the association between plasma *N*-formylmethionine and 28-day mortality (See Additional file 3).

**Table 1:** Demographic and clinical characteristics of study patients

Baseline characteristics	VITdAL-ICU cohort	RoCI cohort
No.	428	90
Age mean (SD)	64.2 (14.9)	55.0 (14.5)
Female sex no. (%)	38 (42)	39 (43)
Non-White race no. (%)	0 (0)	20 (22)
APACHE II mean (SD)	–	25.8 (9.9)
SAPS II mean (SD)	33.4 (15.4)	–
Sepsis no. (%)	31 (7)	51 (63)
Intubation no. (%)	279 (65)	44 (49)
*Vasopressors no. (%)*	201 (48)	26 (29)
ICU		
Anesthesia ICU no. (%)	81 (19)	0 (0)
Cardiac surgery ICU no. (%)	124 (29)	0 (0)
Surgical ICU no. (%)	25 (6)	0 (0)
Medical ICU no. (%)	90 (21)	90 (100)
Neurological ICU no. (%)	106 (25)	0 (0)
Day 0 creatinine mean (SD)	1.40 (1.00)	2.09 (1.98)
*28-Day mortality no. (%)*	95 (22)	30 (33)

**Table 2:** Characteristics of study patients by day 0 *N*-formylmethionine abundance quartiles

Characteristics	*N*-formylmethionine abundance at day 0				*P* value
	Q1	Q2	Q3	Q4	
*VITdAL-ICU cohort*					
No	107	107	107	107	
Age mean (SD)	56.3 (15.3)	62.1 (17.0)	68.1 (11.7)	70.2 (10.7)	< 0.001*
Female sex no. (%)	41 (38)	32 (30)	39 (36)	39 (36)	0.59
Non-White no. (%)	0 (0)	0 (0)	0 (0)	0 (0)	
SAPS II mean (SD)	32.5 (17.0)	32.7 (16.2)	33.7 (13.4)	34.5 (15.0)	0.76*
Sepsis no. (%)	2 (2)	9 (8)	7 (7)	13 (12)	0.033
Creatinine day 0 mean (SD)	0.91 (0.62)	1.05 (0.55)	1.50 (0.86)	2.15 (1.28)	< 0.001*
Procalcitonin ng/ml day 0 median [IQR]	0.14 [0.06, 0.45]	0.20 [0.08, 0.64]	0.62 [0.20, 1.94]	1.24 [0.45, 4.12]	0.001^†^
Propofol no. (%)	27 (25)	28 (26)	25 (27)	24 (23)	0.94
ICU					< 0.009
Anesthesia ICU no. (%)	24 (23)	16 (15)	20 (18)	22 (21)	
Cardiac surgery ICU no. (%)	19 (18)	32 (30)	41 (38)	34 (31)	
Surgical ICU No. (%)	4 (4)	6 (6)	6 (6)	6 (6)	
Medicine ICU no. (%)	19 (18)	22 (21)	20 (18)	30 (28)	
Neurological ICU no. (%)	40 (38)	31 (29)	21 (19)	15 (14)	
28-Day mortality	13 (12)	16 (15)	25 (23)	41 (38)	< 0.001
180-day mortality	24 (22)	25 (23)	43 (40)	63 (59)	< 0.001
*RoCI cohort*					
No	23	22	23	22	
Age mean (SD)	48.5 (16.7)	59.6 (13.0)	56.5 (12.3)	55.5 (14.2)	0.06*
Female sex no. (%)	9 (39)	8 (36)	10 (43)	12 (55)	0.63
Non-White no. (%)	5 (22)	6 (27)	5 (22)	4 (18)	0.54
APACHE II mean (SD)	21.6 (11.6)	25.1 (9.5)	26.0 (9.4)	30.5 (6.8)	0.02*
Sepsis no. (%)	8 (38)	11 (55)	16 (73)	16 (89)	0.007
Creatinine day 0 mean (SD)	0.9 (0.5)	1.2 (0.6)	1.5 (1.0)	2.6 (1.9)	< 0.001*
Procalcitonin ng/ml day 0 median [IQR]^††^	0.21 [0.91, 1.17]	0.48 [0.12, 2.50]	0.70 [0.22, 1.08]	1.08 [0.12, 8.73]	0.46^†^
ICU					
Medicine ICU no. (%)	23 (100)	22 (100)	23 (100)	22 (100)	
28-Day mortality	5 (22)	7 (32)	6 (26)	12 (55)	0.093
180-Day mortality	8 (35)	9 (41)	9 (39)	13 (59)	0.37

Data presented as n (%) unless otherwise indicated

*P* values determined by chi-square unless designated by (*) then P value determined by ANOVA or by (†) determined by Kruskal-Wallis test. ^††^Procalcitonin measured in 53 of the RoCI cohort patients

In day 0 VITdAL-ICU cohort plasma samples, significant differences by t test exist for 517 individual metabolites (*q *value threshold of 0.05) in subjects with elevated *N*-formylmethionine relative to those without. t test results show that 430 metabolites were increased and 87 metabolites decreased in subjects with elevated *N*-formylmethionine relative to those without (See Additional file 4: Day 0 t test VITdAL-ICU tab). Selected plots of normalized unadjusted metabolite data highlight the day 0 increases in short-chain acylcarnitine (C2–C7), branched chain amino acids (BCAA), purine and pentose phosphate pathway metabolites all relative to *N*-formylmethionine quartiles (See Additional file 5). Regarding subject metabolomic profiles, though the multivariable OPLS-DA model has marginal predictability (Q2 = 0.35), the model is valid (*y* axis Q2 intercept of − 0.102) and is significantly different in patients with elevated *N*-formylmethionine compared to those without (CV-ANOVA *p* value < 0.001) (See Additional file 6) [28, 29].

In the VITdAL-ICU cohort linear regression analysis, there were significant associations at day 0 between abundance of 399 individual metabolites (*q *value threshold of 0.05) and increased *N*-formylmethionine abundance (analyzed as continuous) following adjustment for age, sex, SAPS II, admission diagnosis, and baseline 25(OH)D (See Additional file 7: Highlights and Additional file 4: Day 0 VITdAL-ICU tab for full data). Metabolites associated with increased *N*-formylmethionine at day 0 included elevated lactate, short-chain acylcarnitines, as well as BCAA, kynurenine pathway, and pentose phosphate pathway metabolites and decreased lysophosphatidylcholines. The same pattern was observed with further adjustment for bilirubin, creatinine, propofol exposure, or sepsis as well as removal of the SAPS II term (See Additional file 8).

Mixed-effects modeling of 1215 total days 0, 3, and 7 plasma samples from the VITdAL-ICU cohort shows 432 metabolites were significantly positively associated with increased *N*-formylmethionine abundance highlighted by increases in lactate, short-chain acylcarnitines, BCAA, purine and pentose phosphate pathway metabolites (See Additional file 9: Highlights and Additional file 4: Days 0, 3, and 7 VITdAL-ICU tab for full data). One hundred and three metabolites had significant negative associations with increased *N*-formylmethionine abundance, primarily by decreased lysophosphatidylcholines (See Additional file 9: Highlights and Additional file 4: Days 0, 3, and 7 VITdAL-ICU tab for full data). Similar patterns of association are found when the VITdAL-ICU cohort was limited to days 0, 3, and 7 data from the 216 VITdAL-ICU cohort subjects who received placebo (See Additional file 10: Highlights and Additional file 4: Days 0, 3, and 7 VITdAL-ICU Placebo tab for full data).

In the VITdAL-ICU cohort, we investigated *N*-formylmethionine-specific metabolic networks via Gaussian graphical models. The Gaussian graphical model analysis revealed eight *N*-formylmethionine-specific functional modules at day 0 (See Additional file 11). Similar to the day 0 linear regression analyses, short-chain acylcarnitine, BCAA, purine, kynurenine pathway, and pentose phosphate pathway metabolites were prominently featured in the *N*-formylmethionine-specific Gaussian graphical model analysis. Metabolites present in each functional module were all elevated with increased *N*-formylmethionine abundance and had biological or functional similarity.

### RoCI cohort

Baseline characteristics of the 90-subject RoCI cohort were previously published and are again presented in Table 1 [18]. Baseline characteristics differed between subjects stratified by *N*-formylmethionine quartile for APACHE II score, sepsis, and creatinine at day 0 (Table 2). The 28-day mortality of the RoCI cohort was 33%. There was a significantly worse survival with the top quartile of *N*-formylmethionine abundance (log-rank *P* < 0.001, Fig. 1B). Patients with the top quartile of plasma *N*-formylmethionine abundance had a fivefold higher odds of 28-day mortality (OR = 5.1 95%CI 1.4–18.7; *P* = 0.015) following adjustment for age, sex, race, and APACHE II score, compared with the other three quartiles. Further adjustment for bilirubin or creatinine did not materially alter the plasma *N*-formylmethionine mortality associations (See Additional file 3). In the 73 patients who had an existing measurement of plasma mitochondrial DNA levels [11, 30], there was a significant positive correlation between plasma mitochondrial DNA levels and *N*-formylmethionine abundance (Pearson correlation coefficient *r*^2^ = 0.47; *P *value < 0.001).

In day 0 RoCI cohort plasma samples, significant differences by *t *test were found for 127 individual metabolites (*q *value threshold of 0.05) in subjects with elevated *N*-formylmethionine relative to those without (See Additional file 4: Day 0 t test RoCI tab). Day 0 t test differences were present with increased individual short-chain acylcarnitine, BCAA, and pentose phosphate pathway metabolites and decreased lysophosphatidylcholine in subjects with the highest quartile of plasma *N*-formylmethionine abundance relative to the lower quartiles. Similar to the VITdAL-ICU cohort, the multivariable OPLS-DA RoCI cohort model is valid (Q2 intercept of -0.271) and has marginal predictability (Q2 = 0.35), but shows significant differences (CV-ANOVA *P *value < 0.001) in metabolomic profiles at day 0 in patients with highest quartile of plasma *N*-formylmethionine abundance relative to the lower quartiles (See Additional file 6) [28, 29].

In adjusted linear regression analysis of day 0 data from the RoCI cohort, significant differences exist in 82 individual metabolites (*q *value threshold 0.05) with increased *N*-formylmethionine abundance following adjustment for age, sex, race, and APACHE II. Day 0 differences were present with increased individual short-chain acylcarnitine, BCAA, and pentose phosphate pathway species in subjects with increased *N*-formylmethionine abundance (See Additional file 12: Highlights and Additional file 4: Day 0 RoCI for full data). Similar patterns were observed with further adjustment for bilirubin, creatinine, or sepsis (See Additional file 8).

### Linear regression diagnostics

As determined by the variance inflation factor, in both cohorts the independent variables do not share a perfect, linear relationship. The Breusch–Pagan/Cook–Weisberg tests indicate that heteroskedasticity was present in both cohorts, and thus, robust standard errors were utilized in multiple linear regression. Individual metabolites at day 0 were shown to have approximately normal residuals when subjected to multiple linear regression with *N*-formylmethionine as the exposure in both the VITdAL-ICU and RoCI cohorts. Slight deviations from normal were generally noted at the upper tail. The residuals of xenobiotics (i.e., medications) flagrantly violated normality and thus linear regression estimates are not reported. In both cohorts, Cook's distance values were all less than 0.5, indicating that the multiple linear regression associations were not driven by outliers.

### Metabolites common to both VITdAL-ICU and RoCI cohorts

**Fig. 2:**
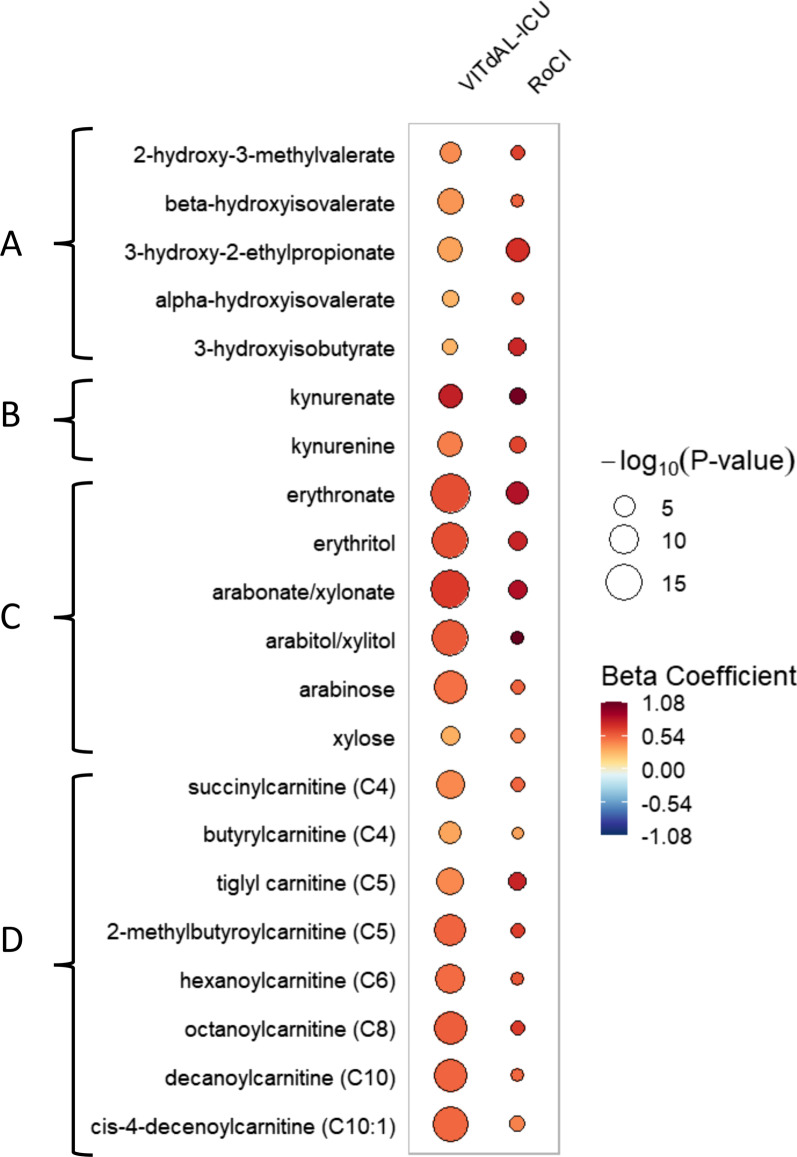
Rain plot of metabolites significantly associated with increased N-formylmethionine in VITdAL-ICU and RoCI cohorts. The correlations between individual metabolites and *N*-formylmethionine abundance at day 0 were determined for the VITdAL-ICU cohort utilizing linear regression models correcting for age, sex, SAPS II, admission diagnosis, and 25(OH)D at day 0. For the RoCI cohort, correlations between individual metabolites and *N*-formylmethionine abundance at day 0 were determined utilizing linear regression models correcting for age, sex, race, and APACHE II score. The magnitude of beta coefficient estimates (effect size) is shown by a color fill scale, and the corresponding significance level (− log_10_(*P *value)) is represented by size of the circle. The intensity of the red or blue fill color represents an increase or decrease, respectively, in effect size for that metabolite relative to *N*-formylmethionine abundance. All respective effect sizes (beta coefficient) and q values can be found in tabular form in Additional file 13. All metabolites shown have a false discovery rate adjusted *p *value (*q *value) < 0.09 in both cohorts. **A** Branched chain amino acid (BCAA) metabolites; **B** kynurenine pathway metabolites; **C** pentose phosphate pathway metabolites; and **D** short- and medium-chain acylcarnitines

In the 337 metabolites analyzed in both the VITdAL-ICU and RoCI cohorts, adjusted linear regression of day 0 plasma shows that with increases in *N*-formylmethionine abundance, 80 metabolites common to both the VITdAL-ICU and RoCI cohorts were changed (*q *value threshold of 0.10) with increases in *N*-formylmethionine abundance. Fifty-five metabolites common to both the VITdAL-ICU and RoCI cohorts had significant changes (*q *value threshold of 0.05) with increases in *N*-formylmethionine abundance. For each of the 55 individual metabolites, the direction of the relationship (increased or decreased) was identical in both cohorts (Table 3 for highlights; Additional file 13: Full data). With increased *N*-formylmethionine abundance, both cohorts had elevated individual short-chain acylcarnitine, BCAA, kynurenine pathway, and pentose phosphate pathway metabolites. The rain plot shows highlighted day 0 metabolites that increased in the VITdAL-ICU and validation cohorts relative to increases in *N*-formylmethionine abundance (Fig. 2). While differences in rain plot circle size reflective of study size exist for the VITdAL-ICU and RoCI cohorts, the effect size and direction of effect followed similar patterns for individual metabolites (Table 3).

**Table 3:** Highlighted metabolites significantly changed at day 0 with increasing *N*-formylmethionine in VITdAL-ICU and RoCI cohorts

Metabolite	VITdAL-ICU cohort		RoCI cohort		Super pathway	Sub pathway
	Beta coefficient	*q* value	Beta coefficient	*q* value		
Beta-hydroxyisovalerate	0.37	1.69 E−07	0.53	3.26 E−02	Amino acid	BCAA metabolism
2-Hydroxy-3-methylvalerate	0.40	3.16 E−05	0.64	2.43 E−02	Amino acid	BCAA metabolism
3-Hydroxy-2-ethylpropionate	0.33	7.36 E−07	0.70	5.40 E−05	Amino acid	BCAA metabolism
3-Hydroxyisobutyrate	0.27	4.39 E−03	0.74	2.54 E−03	Amino acid	BCAA metabolism
Kynurenate	0.77	1.49 E−06	1.04	6.56 E−03	Amino acid	Kynurenine metabolism
Kynurenine	0.44	3.56 E−07	0.63	6.79 E−03	Amino acid	Kynurenine metabolism
Lactate	0.11	5.46 E−02	0.50	3.22 E−02	Carbohydrate	Glycolysis
Succinylcarnitine (C4)	0.41	8.64 E−09	0.52	2.48 E−02	Energy	Short-chain acylcarnitine
Tiglyl carnitine (C5)	0.41	1.18 E−08	0.75	3.68 E−03	Amino acid	Short-chain acylcarnitine
2-Methylbutyroylcarnitine (C5)	0.52	4.99 E−11	0.65	2.38 E−02	Amino acid	Short-chain acylcarnitine
Hexanoylcarnitine (C6)	0.50	7.36 E−10	0.57	6.08 E−02	Lipid	Short-chain acylcarnitine
Arabinose	0.48	2.40 E−12	0.53	2.09 E−02	Carbohydrate	Pentose metabolism
Arabitol/xylitol	0.56	4.77 E−15	1.08	3.65 E−02	Carbohydrate	Pentose metabolism
Arabonate/xylonate	0.66	1.56 E−16	0.86	3.84 E−04	Carbohydrate	Pentose metabolism
Erythritol	0.60	2.53 E−14	0.75	1.05 E−04	Carbohydrate	Pentose metabolism
Erythronate*	0.60	9.57 E−18	0.83	6.49 E−05	Carbohydrate	Pentose metabolism
Xylose	0.29	1.70 E−04	0.44	2.39 E−02	Carbohydrate	Pentose metabolism
7-Methylguanine	0.39	2.82 E−07	0.39	3.52 E−02	Nucleotide	Purine metabolism
Allantoin	0.27	7.06 E−08	0.88	3.66 E−03	Nucleotide	Purine metabolism
1-Methyladenosine	0.43	5.30 E−15	0.35	4.86 E−03	Nucleotide	Purine metabolism
*N*^2^,*N*^2^-Dimethylguanosine	0.73	9.22 E−14	0.91	1.34 E−04	Nucleotide	Purine metabolism
N6-carbamoylthreonyladenosine	0.78	1.32 E−22	0.67	6.62 E−04	Nucleotide	Purine metabolism

For the VITdAL-ICU cohort, significant associations between *N*-formylmethionine abundance and the 983 individual metabolites at day 0 were determined utilizing linear regression correcting for age, sex, baseline 25(OH)D, SAPS II, and admission diagnosis. For the RoCI cohort, significant associations between *N*-formylmethionine abundance and the 411 individual metabolites at day 0 were determined via linear regression correcting for age, sex, ethnicity, and APACHE II utilizing robust standard errors. A false discovery rate adjusted *p *value (*q *value) threshold of 0.05 was used to identify all significant differences. For the short-chain acylcarnitine sub-pathway: a capital C is followed by the number of carbons within the fatty acyl group attached to the carnitine. * indicates putatively annotated compound

## Discussion

With elevated physiologic stress, a metabolic shift in energy production occurs away from mitochondrial beta fatty oxidation to favor glycolysis for energy production, redox balance, and substrate need for biosynthesis [31, 32]. Although previous work suggests that endogenous danger signals are important in the critical illness response, our robust data argue that distinct nuanced energy metabolism patterns, and elevated mortality risks are present with increased *N*-formylmethionine abundance [11, 33]. We identified *N*-formylmethionine-specific metabolite networks using Gaussian graphical models which highlight the same groups identified in our regression analyses [34]. The analyses in the VITdAL-ICU cohort and confirmed in the RoCI cohort highlight the potential of *N*-formylmethionine as a metabolic shift biomarker reflective of cellular performance and mortality outcome.

Though the response to critical illness is complex and requires highly demanding cellular functions, the initial signals of physiological stress are crude and widely conserved [35]. Due to evolutionary resemblance, mitochondrial DNA and N-formyl-methionyl peptides act similarly to bacterial pathogen-associated molecular patterns [36, 37]. Circulating *N*-formylmethionine in critical illness is produced by the degradation of mitochondrial N-formyl-methionyl peptides and is a marker for mitochondrial-based protein turnover [13, 38]. Though not required for activation of N-formylpeptide receptor (FPR) subtypes, we argue that the abundance of *N*-formylmethionine is a biomarker of cell stress and signals ongoing disruption of homeostasis [39].

**Fig. 3:**
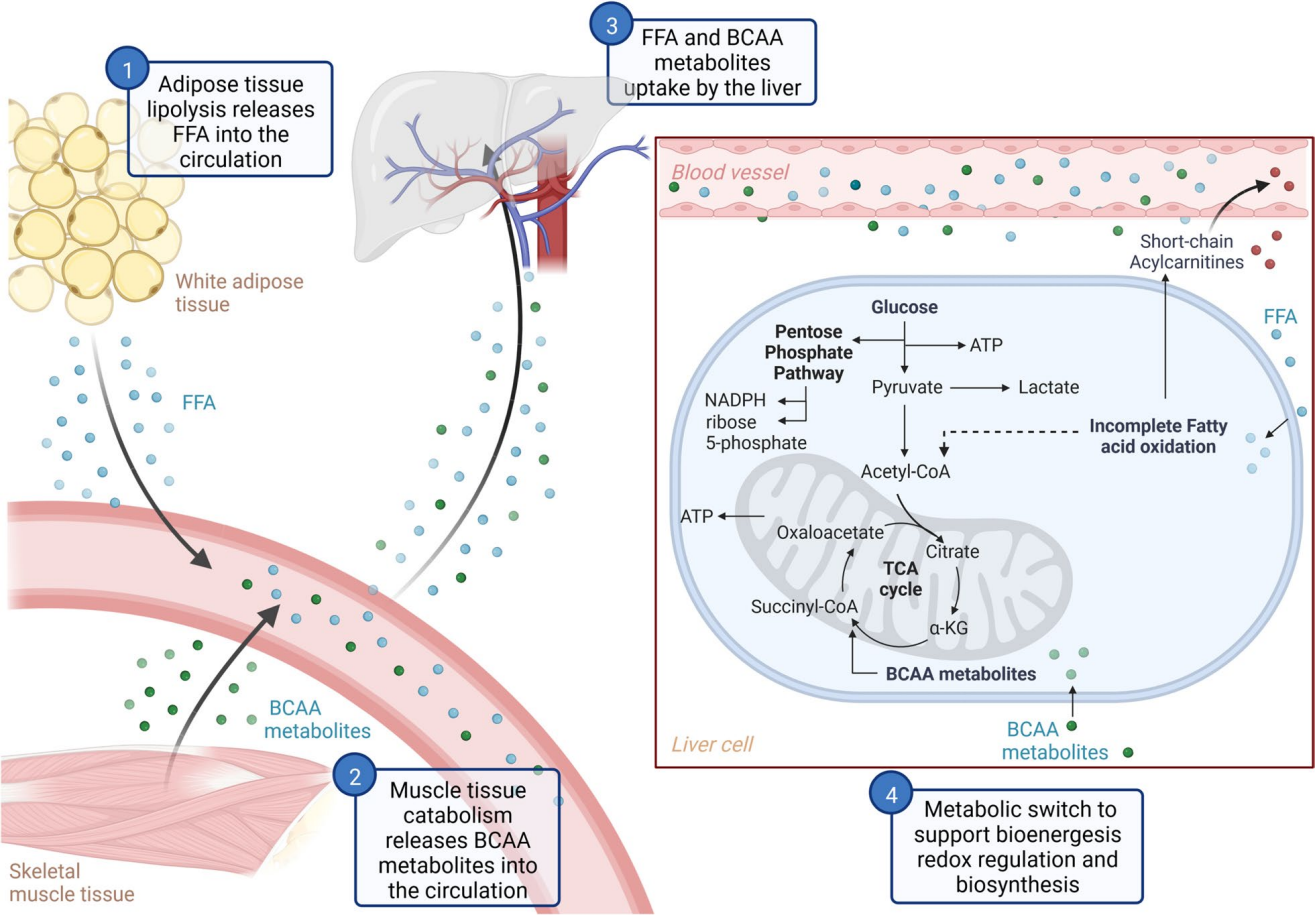
Energy utilization pathways during cell stress. In response to cell stress, adipose and muscle tissue catabolism releases free fatty acids (FAA) and branch chain amino acid metabolites (BCAA) into circulation (steps 1 and 2). Free fatty acids and branch chain amino acid metabolites are taken up by the liver (step 3). Metabolic switch including utilization of branch chain amino acid metabolites in the TCA cycle, pentose phosphate pathway activation, and incomplete fatty acid oxidation resulting in energy production, redox regulation, biosynthesis, and production of circulating short-chain acylcarnitines

In both cohorts, we found evidence of a metabolic shift (Fig. 3). First, the observed rise in lactate with increased *N*-formylmethionine abundance indicates an increase in aerobic glycolysis or tissue hypoxia [40]. Second, we show that with increased *N*-formylmethionine abundance, both cohorts have significant increases in modified purine nucleosides known to be liberated during oxidative stress [41]. Third, our observation of increased circulating plasma short-chain acylcarnitines (C2–C7) with increased *N*-formylmethionine abundance is evidence of incomplete mitochondrial fatty acid β-oxidation and reflects a decrease in mitochondrial energy production [42]. Fourth, our data show that circulating BCAA metabolites are elevated with increased *N*-formylmethionine abundance. BCAA metabolites are known to be utilized for energy production when mitochondrial fatty acid β-oxidation is incomplete [43]. Further, the observed elevated C3- and C5-acylcarnitines are known to be derived from BCAA metabolites [44]. In our study cohorts, the observed increases in plasma lactate, purine nucleotides, short-chain acylcarnitines, and BCAA catabolic metabolites with increased *N*-formylmethionine abundance are thus indicative of a metabolic shift.

Our results show novel increases in pentose phosphate pathway metabolites with increased *N*-formylmethionine abundance. The pentose phosphate pathway is important for redox balance via NADPH production through a oxidative branch as well as nucleic acid synthesis through a non-oxidative branch (See Additional file 14) [45]. Though quiescent at homeostasis and rarely reported in critical illness, evidence suggests that the pentose phosphate pathway acts as a “metabolic redox sensor” that is upregulated during oxidative stress [46, 47]. Transcriptomic profiling of whole blood in critically ill patients has shown increases in pentose phosphate pathway oxidative branch activity, suggesting a metabolic shift away from mitochondrial beta oxidation [48]. We show that with increased *N*-formylmethionine abundance, both cohorts have evidence of significant increases in the pentose phosphate pathway metabolites of the non-oxidative branch, which is further indicative of a metabolic shift (Table 3).

Our novel study approach has several strengths. Both of our cohorts were analyzed using the same Metabolon platform. Further, our use of clinical data allows for modeling of metabolite abundance with adjustment for subject characteristics. Our findings are consistent with known metabolic shift patterns increasing the biological plausibility of our work. Finally, we validated our findings in an external critical illness cohort.

There are potential limitations to our approach. Despite our RoCI cohort being diverse, our VITdAL-ICU cohort subjects are from a white population. Our ability to determine causality is limited as we performed two metabolomics cohort studies. Our approach is subject to bias and confounding despite our covariate and multiplicity adjustment. With only 337 metabolites common to both cohorts, some important individual metabolites noted in the VITdAL-ICU cohort cannot be analyzed in the RoCI cohort. As we did not measure the *N*-formylmethionine by concentration, our abundance quartiles are unlikely to have the same *N*-formylmethionine concentration in both the VITdAL-ICU and RoCI cohorts. Thus, our comparison of top quartiles of abundance between the two cohorts cannot provide an accurate cut point for the metabolic shift responses to cell stress. Further, we do not have extensive data on nutritional support which may confound the metabolite differences observed. Finally, our similar metabolic shift findings in our two cohorts should be considered as a hypothesis that requires further confirmation and careful interpretation.

Metabolomics can bridge the knowledge gap between clinical heterogeneity and severity of critical illness [49]. Finding consistent metabolic shift patterns in clinically heterogeneous cohorts has the potential to identify biologically meaningful phenotypes [50]. We find that with severe cell stress indicated by elevated circulating *N*-formylmethionine, a shared metabolic shift exists in both the VITdAL-ICU and RoCI cohorts despite clinical heterogeneity. As different clinical presentations can produce different metabolomic patterns at different times, the finding of a shared metabolic shift associated with cell stress may be helpful in detecting a severity of illness phenotype [51]. Next research steps include confirming our metabolic profiling and mortality results with determination of *N*-formylmethionine concentrations via ELISA [52]. To be generalizable and robust, such further work will require multiple cohorts with clinical heterogeneity and adequate sample sizes. If confirmed, such pathobiology-driven study of the clinical heterogeneity of severe critical illness has future clinical implications in providing insights beyond the limitations of illness severity scores and outcome prediction models [53].

## Conclusions

Our findings offer a deep and nuanced dive into the early critical illness response. In addition to elevated mortality, we found that patients with elevated *N*-formylmethionine have increases in metabolites related to short-chain acylcarnitines, BCAA catabolism, and the pentose phosphate pathway. The metabolomic patterns related to energy utilization with increases in *N*-formylmethionine allow for a common metabolomic shift to be identified in the heterogeneous critically ill population. Defining a common metabolic shift is a crucial first step toward a structural framework to increase mechanistic understanding of early critical illness.

## Supplementary Information

**Additional file 1.** Supplemental Methods.

**Additional file 2.** Admission Diagnosis category of VITdAL-ICU cohort patients by day 0 N-Formylmethionine abundance quartiles.

**Additional file 3.** Associations between plasma N-formylmethionine abundance quartiles and 28-day mortality. Logistic regression models shown for crude and adjusted VITdAL-ICU and RoCI cohort survival data. Additional models shown with adjustment for total bilirubin, creatinine, and/or propofol exposure.

**Additional file 4.** Full metabolomics results from VITdAL-ICU and RoCI Cohorts. Six Individual Worksheets include Day 0 t test VITdAL-ICU: t test results of Day 0 data from the VITdAL-ICU cohort; Day 0 VITdAL-ICU: Linear regression results of Day 0 data from the VITdAL-ICU cohort adjusted for age, sex, baseline 25(OH)D, SAPS II, and admission diagnosis; Day 0,3,7 VITdAL-ICU: Mixed effects regression results of Day 0,3,7 data from the VITdAL-ICU cohort adjusted for age, sex, baseline 25(OH)D, absolute increase in 25(OH)D at day 3, SAPS II, plasma day, admission diagnosis, and an individual subject-specific random-intercept; Day 0,3,7 VITdAL-ICU Placebo: Mixed effects regression results of Day 0,3,7 data from the VITdAL-ICU cohort in patients who received Placebo adjusted for age, sex, baseline 25(OH)D, absolute increase in 25(OH)D at day 3, SAPS II, plasma day, admission diagnosis, and an individual subject-specific random-intercept; Day 0 t test RoCI: t test results of Day 0 data from the RoCI cohort; Day 0 RoCI: Linear regression results of Day 0 data from the RoCI cohort adjusted for age, sex, race, and APACHE II score. T-statistics are presented for t tests. Beta coefficients are presented for linear regression and mixed effects analysis. In all worksheets, raw p values are presented. A false discovery rate adjusted p value (q value) threshold of 0.05 was used to identify all significant differences.

**Additional file 5.** Dot Boxplots of metabolite Sub Pathways by N-formylmethionine quartiles at Day 0. Unadjusted normalized abundance of metabolites from 428 VITdAL-ICU cohort subjects at day 0 by quartiles of N-formylmethionine abundance. Sub Pathways shown include short-chain acylcarnitine, branched chain amino acid, pentose phosphate pathway and purine metabolites. Box plots show the data between the first and third quartile (box), the median and whiskers at 1.5 times the interquartile range. All individual data points are visualized using a bee swarm plot.

**Additional file 6.** Day 0 OPLS-DA model goodness of fit, predictive ability and model significance in both VITdAL-ICU and RoCI Cohorts. R2 and Q2 quality metrics, the permutation diagnostics and overall model significance are presented.

**Additional file 7.** Highlighted Significantly Different Metabolites with increased N-formylmethionine abundance in the VITdAL-ICU Cohort at day 0.

**Additional file 8.** Additional metabolomics results from VITdAL-ICU and RoCI Cohorts. Nine individual worksheets include Linear regression results of Day 0 data from the VITdAL-ICU cohort adjusted for age, sex, baseline 25(OH)D, SAPS II, admission diagnosis, with additional adjustment for total bilirubin, creatinine, sepsis or propofol exposure. Day 0 data from the VITdAL-ICU cohort is also shown adjusted for age, sex, baseline 25(OH)D, and admission diagnosis but without SAPS II. Linear regression results of Day 0 data from the RoCI cohort are shown adjusted for age, sex, baseline 25(OH)D, SAPS II, admission diagnosis, with additional adjustment for total bilirubin, creatinine, or sepsis. In all worksheets, raw p values are presented. A false discovery rate adjusted p value (q value) threshold of 0.05 was used to identify all significant differences.

**Additional file 9.** Metabolites significantly changed with increased N-formylmethionine abundance in the VITdAL-ICU Cohort over days 0, 3 and 7.

**Additional file 10.** Metabolites significantly changed with increased N-formylmethionine abundance in VITdAL-ICU Cohort placebo patients over days 0, 3 and 7.

**Additional file 11.** Day 0 N-formylmethionine-specific Metabolic Networks with similar effects via Gaussian graphical models.

**Additional file 12.** Significantly Different Metabolites with increased N-formylmethionine abundance in the RoCI Cohort at day 0.

**Additional file 13.** Significantly Different Metabolites with increased N-formylmethionine at day 0 in both VITdAL-ICU and RoCI Cohorts.

**Additional file 14.** Increased Pentose Phosphate Pathway Metabolites with N-formylmethionine. Metabolites increased in the VITdAL-ICU and RoCI cohorts with increased N-formylmethionine abundance are highlighted in red, metabolites increased in the VITdAL-ICU cohort with increased N-formylmethionine abundance are highlighted in green. The Pentose Phosphate Pathway produces NADPH and ribose 5-phosphate for redox regulation and biosynthesis, respectively.

## Data Availability

The datasets supporting the conclusions of this article are included within the article and its additional files.

## Abbreviations

DAMPs: Danger-associated molecular patterns
ICU: Intensive Care Unit
OR: Odds ratio
VITdAL-ICU trial: Correction of Vitamin D Deficiency in Critically Ill Patients trial
25(OH)D: 25-Hydroxyvitamin D
RoCI: The Registry of Critical Illness
OPLS-DA: Orthogonal partial least square-discriminant analysis
BCAA: Branched chain amino acid
GGM: Gaussian graphical models
APACHE: Acute Physiology and Chronic Health Evaluation
SAPS: Simplified Acute Physiology Score
CV-ANOVA: Analysis of variance of the cross-validated residuals

## References

[1] Scicluna BP (2022). Resolving patient heterogeneity in critical illness requires multi-scale approaches. EBioMedicine.

[2] Braga D, Barcella M, Herpain A, Aletti F, Kistler EB, Bollen Pinto B, Bendjelid K, Barlassina C (2019). A longitudinal study highlights shared aspects of the transcriptomic response to cardiogenic and septic shock. Crit Care.

[3] Amrein K, Lasky-Su JA, Dobnig H, Christopher KB (2021). Metabolomic basis for response to high dose vitamin D in critical illness. Clin Nutr.

[4] Initiative C-H (2021). Mapping the human genetic architecture of COVID-19. Nature.

[5] Frishberg A, Kooistra E, Nuesch-Germano M, Pecht T, Milman N, Reusch N, Warnat-Herresthal S, Bruse N, Handler K, Theis H (2022). Mature neutrophils and a NF-kappaB-to-IFN transition determine the unifying disease recovery dynamics in COVID-19. Cell Rep Med.

[6] Carre JE, Orban JC, Re L, Felsmann K, Iffert W, Bauer M, Suliman HB, Piantadosi CA, Mayhew TM, Breen P (2010). Survival in critical illness is associated with early activation of mitochondrial biogenesis. Am J Respir Crit Care Med.

[7] Rautanen A, Mills TC, Gordon AC, Hutton P, Steffens M, Nuamah R, Chiche JD, Parks T, Chapman SJ, Davenport EE (2015). Genome-wide association study of survival from sepsis due to pneumonia: an observational cohort study. Lancet Respir Med.

[8] Russell CD, Baillie JK (2017). Treatable traits and therapeutic targets: Goals for systems biology in infectious disease. Curr Opin Syst Biol.

[9] Chen GY, Nunez G (2010). Sterile inflammation: sensing and reacting to damage. Nat Rev Immunol.

[10] Nakahira K, Hisata S, Choi AM (2015). The roles of mitochondrial damage-associated molecular patterns in diseases. Antioxid Redox Signal.

[11] Johansson PI, Nakahira K, Rogers AJ, McGeachie MJ, Baron RM, Fredenburgh LE, Harrington J, Choi AMK, Christopher KB (2018). Plasma mitochondrial DNA and metabolomic alterations in severe critical illness. Crit Care.

[12] Faust HE, Reilly JP, Anderson BJ, Ittner CAG, Forker CM, Zhang P, Weaver BA, Holena DN, Lanken PN, Christie JD (2020). Plasma mitochondrial DNA levels are associated with ARDS in trauma and sepsis patients. Chest.

[13] Raabe CA, Groper J, Rescher U (2019). Biased perspectives on formyl peptide receptors. Biochim Biophys Acta Mol Cell Res.

[14] Johnson CH, Ivanisevic J, Siuzdak G (2016). Metabolomics: beyond biomarkers and towards mechanisms. Nat Rev Mol Cell Biol.

[15] Amrein K, Schnedl C, Holl A, Riedl R, Christopher KB, Pachler C, Urbanic Purkart T, Waltensdorfer A, Munch A, Warnkross H (2014). Effect of high-dose vitamin D3 on hospital length of stay in critically ill patients with vitamin D deficiency: the VITdAL-ICU randomized clinical trial. JAMA.

[16] Chary S, Amrein K, Lasky-Su J, Dobnig H, Christopher KB (2021). The sex-specific metabolic response to critical illness: a post-hoc metabolomics study of the VITdAL-ICU trial. Sci Rep.

[17] Dolinay T, Kim YS, Howrylak J, Hunninghake GM, An CH, Fredenburgh L, Massaro AF, Rogers A, Gazourian L, Nakahira K (2012). Inflammasome-regulated cytokines are critical mediators of acute lung injury. Am J Respir Crit Care Med.

[18] Rogers AJ, McGeachie M, Baron RM, Gazourian L, Haspel JA, Nakahira K, Fredenburgh LE, Hunninghake GM, Raby BA, Matthay MA (2014). Metabolomic derangements are associated with mortality in critically ill adult patients. PLoS ONE.

[19] Chong J, Xia J (2020). Using MetaboAnalyst 4.0 for metabolomics data analysis, interpretation, and integration with other omics data. Methods Mol Biol.

[20] Benjamini Y, Hochberg Y (1995). Controlling for false discovery rate: a practical and powerful approach to multiple testing. J R Stat Soc Ser B (Methodol).

[21] Matthew B, William R (2003). Partial least squares for discrimination. J Chemom.

[22] Worley B, Powers R (2016). PCA as a practical indicator of OPLS-DA model reliability. Curr Metab.

[23] StataCorp: Stata Statistical Software: Release 16. In*.* College Station, TX: StataCorp LP (2019)

[24] Henglin M, Niiranen T, Watrous JD, Lagerborg KA, Antonelli J, Claggett BL, Demosthenes EJ, von Jeinsen B, Demler O, Vasan RS (2019). A single visualization technique for displaying multiple metabolite-phenotype associations. Metabolites.

[25] Zhang B, Tian Y, Zhang Z (2014). Network biology in medicine and beyond. Circ Cardiovasc Genet.

[26] Krumsiek J, Suhre K, Illig T, Adamski J, Theis FJ (2011). Gaussian graphical modeling reconstructs pathway reactions from high-throughput metabolomics data. BMC Syst Biol.

[27] Do KT, Pietzner M, Rasp DJ, Friedrich N, Nauck M, Kocher T, Suhre K, Mook-Kanamori DO, Kastenmuller G, Krumsiek J (2017). Phenotype-driven identification of modules in a hierarchical map of multifluid metabolic correlations. NPJ Syst Biol Appl.

[28] Westerhuis JA, Hoefsloot HCJ, Smit S, Vis DJ, Smilde AK, van Velzen EJJ, van Duijnhoven JPM, van Dorsten FA (2008). Assessment of PLSDA cross validation. Metabolomics.

[29] Eriksson L, Trygg J, Wold S (2008). CV-ANOVA for significance testing of PLS and OPLS models. J Chemom.

[30] Nakahira K, Kyung SY, Rogers AJ, Gazourian L, Youn S, Massaro AF, Quintana C, Osorio JC, Wang Z, Zhao Y (2013). Circulating mitochondrial DNA in patients in the ICU as a marker of mortality: derivation and validation. PLoS Med.

[31] Preiser JC, Ichai C, Orban JC, Groeneveld AB (2014). Metabolic response to the stress of critical illness. Br J Anaesth.

[32] Puthucheary ZA, Astin R, McPhail MJW, Saeed S, Pasha Y, Bear DE, Constantin D, Velloso C, Manning S, Calvert L (2018). Metabolic phenotype of skeletal muscle in early critical illness. Thorax.

[33] Venereau E, Ceriotti C, Bianchi ME (2015). DAMPs from cell death to new life. Front Immunol.

[34] Mittelstrass K, Ried JS, Yu Z, Krumsiek J, Gieger C, Prehn C, Roemisch-Margl W, Polonikov A, Peters A, Theis FJ (2011). Discovery of sexual dimorphisms in metabolic and genetic biomarkers. PLoS Genet.

[35] Jacob F (1970) La Logique du vivant. Une histoire de l’hérédité. Paris: Éditions Gallimard

[36] Hemmi H, Takeuchi O, Kawai T, Kaisho T, Sato S, Sanjo H, Matsumoto M, Hoshino K, Wagner H, Takeda K (2000). A Toll-like receptor recognizes bacterial DNA. Nature.

[37] Waller JP (1963). The Nh2-terminal residues of the proteins from cell-free extracts of *E. coli*. J Mol Biol.

[38] Garcia-Contreras M, Tamayo-Garcia A, Pappan KL, Michelotti GA, Stabler CL, Ricordi C, Buchwald P (2017). Metabolomics study of the effects of inflammation, hypoxia, and high glucose on isolated human pancreatic islets. J Proteome Res.

[39] Gao JL, Becker EL, Freer RJ, Muthukumaraswamy N, Murphy PM (1994). A high potency nonformylated peptide agonist for the phagocyte *N*-formylpeptide chemotactic receptor. J Exp Med.

[40] Levy B, Desebbe O, Montemont C, Gibot S (2008). Increased aerobic glycolysis through beta2 stimulation is a common mechanism involved in lactate formation during shock states. Shock.

[41] Seymour CW, Yende S, Scott MJ, Pribis J, Mohney RP, Bell LN, Chen YF, Zuckerbraun BS, Bigbee WL, Yealy DM (2013). Metabolomics in pneumonia and sepsis: an analysis of the GenIMS cohort study. Intensive Care Med.

[42] Koves TR, Ussher JR, Noland RC, Slentz D, Mosedale M, Ilkayeva O, Bain J, Stevens R, Dyck JR, Newgard CB (2008). Mitochondrial overload and incomplete fatty acid oxidation contribute to skeletal muscle insulin resistance. Cell Metab.

[43] Neinast M, Murashige D, Arany Z (2019). Branched chain amino acids. Annu Rev Physiol.

[44] Newgard CB (2012). Interplay between lipids and branched-chain amino acids in development of insulin resistance. Cell Metab.

[45] Kletzien RF, Harris PK, Foellmi LA (1994). Glucose-6-phosphate dehydrogenase: a "housekeeping" enzyme subject to tissue-specific regulation by hormones, nutrients, and oxidant stress. FASEB J.

[46] Kruger A, Gruning NM, Wamelink MM, Kerick M, Kirpy A, Parkhomchuk D, Bluemlein K, Schweiger MR, Soldatov A, Lehrach H (2011). The pentose phosphate pathway is a metabolic redox sensor and regulates transcription during the antioxidant response. Antioxid Redox Signal.

[47] Haji-Michael PG, Ladriere L, Sener A, Vincent JL, Malaisse WJ (1999). Leukocyte glycolysis and lactate output in animal sepsis and ex vivo human blood. Metabolism.

[48] Nalos M, Parnell G, Robergs R, Booth D, McLean AS, Tang BM (2016). Transcriptional reprogramming of metabolic pathways in critically ill patients. Intensive Care Med Exp.

[49] Thistlethwaite LR, Li X, Burrage LC, Riehle K, Hacia JG, Braverman N, Wangler MF, Miller MJ, Elsea SH, Milosavljevic A (2022). Clinical diagnosis of metabolic disorders using untargeted metabolomic profiling and disease-specific networks learned from profiling data. Sci Rep.

[50] Patti GJ, Yanes O, Siuzdak G (2012). Innovation: Metabolomics: the apogee of the omics trilogy. Nat Rev Mol Cell Biol.

[51] Neugebauer S, Giamarellos-Bourboulis EJ, Pelekanou A, Marioli A, Baziaka F, Tsangaris I, Bauer M, Kiehntopf M (2016). Metabolite profiles in sepsis: developing prognostic tools based on the type of infection. Crit Care Med.

[52] Duvvuri B, Baddour AA, Deane KD, Feser ML, Nelson JL, Demoruelle MK, Lood C (2021). Mitochondrial *N*-formyl methionine peptides associate with disease activity as well as contribute to neutrophil activation in patients with rheumatoid arthritis. J Autoimmun.

[53] Desai N, Gross J (2019). Scoring systems in the critically ill: uses, cautions, and future directions. BJA Educ.

