# Supplementary material for: Circulating N-formylmethionine and metabolic shift in critical illness: a multicohort metabolomics study
Source: Crit Care. 2022 Oct 19;26:321. doi: 10.1186/s13054-022-04174-y (PMC9580206; doi:10.1186/s13054-022-04174-y)
Supplement: Supplementary file 1 — Additional file 1. Supplemental Methods. [file 13054_2022_4174_MOESM1_ESM.docx]

**Additional file 1**

**Supplementary Methods**

Circulating N-formylmethionine and Metabolic Shift in Critical Illness: a multicohort metabolomics study

Martin Ingi Sigurdsson, Hirotada Kobayashi, Karin Amrein, Kiichi Nakahira, Angela J. Rogers, Mayra Pinilla-Vera, Rebecca M. Baron, Laura E. Fredenburgh, Jessica A. Lasky-Su, Kenneth B. Christopher

**VITdAL-ICU Cohort Details:** The VITdAL-ICU trial randomized 475 critically ill adult subjects with 25(OH)D < 20 ng/mL to vitamin D_3_ or placebo given orally or via nasogastric tube once at a dose of 540,000 IU followed by 90,000 IU monthly [1]. The trial was conducted at the University Hospital Graz in Southeast Austria in 5 Medical and Surgical Intensive Care Units. Patients were randomized 1:1 with randomization block size of 8 stratified via ICU type and sex. The primary study outcome was length of hospital stay. Secondary outcomes included 28-day mortality, hospital mortality, 6-month mortality, length of ICU stay as well as 25-hydroxyvitamin D (25(OH)D) levels at day 0, 3, and 7. Blood samples were collected on days 0 (pre-randomization), 3, and 7. Plasma was fractionated, aliquoted, and stored at -70°C. 453 trial subjects had frozen plasma available for analysis. The VITdAL-ICU trial was approved by the institutional ethical committee of the Medical University of Graz and the Austrian Agency for Health and Food Safety. In accordance with Austrian and European Union requirements and the principles of the Declaration of Helsinki, [2] at VITdAL-ICU trial enrollment, written informed consent was obtained, if possible, directly from the patient or from a legal surrogate [1]. Consent included permission for plasma specimens to be saved for future research studies. The post-hoc study research protocol was approved by the Mass General Brigham Human Research Committee Institutional Review Board at the Brigham and Women’s Hospital.

Clinical trial data utilized included age, sex, admission diagnosis category, baseline 25(OH)D, intervention status (placebo vs high dose vitamin D_3_), absolute change in 25(OH)D level at day 3 relative to day 0, and the Simplified Acute Physiology Score (SAPS) II [3] at day 0. 25(OH)D levels were measured in the VITdAL-ICU cohort by chemiluminescence immunoassay [1]. Admission diagnosis category was determined at ICU admission by trial investigators as Neurosurgery, Cardiac surgery, Cardiovascular, Gastrointestinal/liver, Hematologic/Oncology/ Metabolic, Neurologic, Other non-operative, Other operative, Renal, Respiratory, Sepsis/infectious, Thoracic Surgery, Transplantation, Trauma, and Vascular Surgery.

**RoCI Cohort Details:** The Brigham and Women’s Hospital (BWH) Registry of Critical Illness (RoCI) is approved by the Partners IRB committee. The protocol for recruitment for RoCI has been published in detail elsewhere [4]. Briefly, adult patients (over age 18) who are admitted to the BWH Medical Intensive Care Unit are eligible for inclusion in the RoCI within 72 hours of presentation, unless certain exclusion criteria are met (unable to provide consent due to cognitive dysfunction or no appropriate health care proxy, prior refusal, admission purely for comfort care, Jehovah’s Witness status, or a baseline hemoglobin <8 g/dL or hemoglobin <9 g/dL with either admission for active bleeding or with acute ischemia). Prior to RoCI enrollment, written informed consent was obtained which included permission for plasma samples to be utilized and saved for research purposes in the future. Plasma is obtained on days 0, 3, and 7 of enrollment. Extensive phenotypic data (including age, sex, race, key comorbidities, and APACHE II score), laboratory and radiologic data were recorded for all subjects. Classification of Systemic Inflammatory Response (SIRS), Sepsis, and ARDS is determined by a consensus panel of ICU physicians using the current disease classification [5, 6].

All patients in the RoCI cohort had 180-day follow up outcome data available for review. Mortality was determined on the complete cohort using hospital records and the Social Security Administration Death Master File [7]. Race was either self-determined or designated by a patient representative/healthcare proxy. Classification of SIRS, sepsis, and, ARDS was determined by a consensus panel of ICU physicians using the Consensus Conference classifications in existence at the time of enrollment [5, 6]. Acute physiology and chronic health evaluation (APACHE) II score was determined at 24 hours post-ICU admission [8]. Among 225 RoCI subjects described by Dolinay et.al. [4], 90 subjects were selected for metabolic profiling based in part on IL-18 levels for a study of metabolomic signatures of critical illness outcome: 29 with SIRS, 30 with Sepsis, and 31 with sepsis-induced ARDS [9]. Cell-free plasma ND1 mitochondrial DNA levels were previously determined on 73 RoCI cohort patients [10] [11]. Plasma procalcitonin levels was measured using the Procalcitonin Human ELISA Kit (ab100630; Abcam) on 53 RoCI cohort patients [10]. We have previously published metabolomics studies on this 90 subject subset of the RoCI cohort [11-13].

**Sample Preparation:** Blood samples were drawn and transferred into EDTA coated blood collection tubes within 24 hours from study inclusion and processed within 4 hours after venipuncture. Subsequently, plasma was fractionated, aliquoted and stored at -80°C [4]. For the VITdAL-ICU and RoCI cohorts, VITdAL-ICU trial or RoCI cohort subject 150 μl plasma aliquots, respectively, were separately shipped at different times on dry-ice to Metabolon, Inc. Following receipt, the frozen plasma samples were immediately stored at -80^o^C. Metabolites are noted by other investigators to be stable for at least two freeze thaw cycles [14]. To generate metabolomic data for the VITdAL-ICU cohort, a total of 1215 VITdAL-ICU trial plasma samples from 428 subjects at day 0, 413 subjects at day 3, and 374 subjects at day 7 were prepared and analyzed in 2017 [1, 15, 16]. The metabolomic data for the RoCI cohort was produced from a total of 90 RoCI cohort plasma samples from 90 subjects at day 0 that were prepared and analyzed in 2011 [9].

Plasma sample preparation was performed with the automated MicroLab STAR® Liquid Handling system (Hamilton Company, NV, USA). Before extraction, samples were fortified with recovery standards for quality control (QC) purposes. To remove protein, dissociate small molecules bound to protein or trapped in the precipitated protein matrix, and to recover chemically diverse metabolites, proteins were precipitated with methanol via 2 minutes of robust shaking (GenoGrinder 2000 SPEX SamplePrep, NJ, USA) and subsequent centrifugation. The resulting extract was divided into five fractions: two for analysis by two separate reverse phase (RP)/ UPLC-MS/MS methods with positive ion mode electrospray ionization (ESI), one for analysis by RP/UPLC-MS/MS with negative ion mode ESI, one for analysis by HILIC/UPLC-MS/MS with negative ion mode ESI, and one sample was reserved for backup. Samples were placed on a TurboVap® (Zymark, MA, USA) to remove the organic solvent and stored overnight under nitrogen before preparation for analysis.

**Quality Assurance (QA) and Quality Control (QC):**  Several types of controls were utilized with the plasma samples analysis allowing for instrument performance monitoring and aided chromatographic alignment: a pooled matrix sample generated by taking a small volume of each experimental sample served as a technical replicate throughout the data set [17]; extracted water samples served as process blanks [18]; and a cocktail of QC standards that were carefully chosen not to interfere with the measurement of endogenous compounds were spiked into every analyzed sample [19]. Instrument variability was determined by calculating the median relative standard deviation (RSD) for the standards that were added to each sample prior to injection into the mass spectrometers [20]. Overall process variability was determined by calculating the median RSD for all endogenous metabolites (i.e., non-instrument standards) present in 100% of the pooled matrix samples. Experimental samples were randomized across the platform run with QC samples spaced evenly among the injections.

**Ultrahigh Performance Liquid Chromatography-Tandem Mass Spectroscopy (UPLC-MS/MS):**  All methods utilized a Waters ACQUITY ultra-performance liquid chromatography (UPLC) (Waters, MA, USA) and for untargeted lipidomic analysis a Thermo Scientific Q Exactive™ high resolution/accurate mass spectrometer interfaced with a heated electrospray ionization (HESI-II) source and Orbitrap™ mass analyzer operated at 35,000 mass resolution (ThermoFisher Scientific, MA, USA) [21]. The sample extract was dried then reconstituted in solvents compatible to each of the four methods. Each reconstitution solvent contained a series of standards at fixed concentrations to ensure injection and chromatographic consistency. One aliquot was analyzed using acidic positive ion conditions, chromatographically optimized for more hydrophilic compounds. In this method, the extract was gradient eluted from a C18 column (Waters UPLC BEH C18-2.1x100 mm, 1.7 µm) using water and methanol, containing 0.05% perfluoropentanoic acid (PFPA) and 0.1% formic acid (FA). Another aliquot was also analyzed using acidic positive ion conditions; however, it was chromatographically optimized for more hydrophobic compounds [22, 23]. In this method, the extract was gradient eluted from the same C18 column using methanol, acetonitrile, water, 0.05% PFPA, and 0.01% FA and was operated at an overall higher organic content. Another aliquot was analyzed using basic negative ion optimized conditions using a separate dedicated C18 column. The basic extracts were gradient eluted from the column using methanol and water, however with 6.5mM Ammonium Bicarbonate at pH 8. The fourth aliquot was analyzed via negative ionization following elution from a HILIC column (Waters UPLC BEH Amide 2.1x150 mm, 1.7 µm) using a gradient consisting of water and acetonitrile with 10mM Ammonium Formate, pH 10.8. The MS analysis alternated between MS and data-dependent MS^n^ scans using dynamic exclusion [24]. The scan range for both ionization modes was 70–1000 *m/z* [25].

**Data Extraction and Compound Identification:** Raw data was extracted, peak-identified and QC processed using Metabolon’s hardware and software. Compounds were identified by comparison to library entries of purified standards or recurrent unknown entities. Metabolon maintains a library based on authenticated standards that contains the retention time/index (RI), mass-to-charge ratio (*m/z*), and chromatographic data (including MS/MS spectral data) on all molecules present in the library. Furthermore, biochemical identifications are based on three criteria: retention index within a narrow RI window of the proposed identification, accurate mass match to the library +/- 10 ppm, and the MS/MS forward and reverse scores between the experimental data and authentic standards [26]. The MS/MS scores are based on a comparison of the ions present in the experimental spectrum to the ions present in the library spectrum. While there may be similarities between these molecules based on one of these factors, the use of all three data points can be utilized to distinguish and differentiate biochemicals [27]. More than 3300 commercially available purified standard compounds have been acquired and registered into the Metabolon Laboratory Information Management System (LIMS) system for analysis on all platforms for determination of their analytical characteristics. The identification level reported in our tables follows the criteria described by Sumner et al. [28]. Level 1 is a validated identification which confirms a structure with a minimum of two independent and orthogonal data from a pure reference standard under identical analytical conditions. Predictive or externally acquired structure evidence when a reference standard does not exist, (i.e., MS/MS data, exhibiting diagnostic fragments or neutral losses consistent with a specific structure) is a putative identification (Level 2) [29]. Compounds labelled with “*” have identification Level 2. If no label is applied, the identification Level is 1. Compounds labelled with “( )” or “[ ]” indicate a structural isomer of another compound in the spectral library; for example, a steroid that may be sulfated at one of several positions that are indistinguishable by the mass spectrometry data or a diacylglycerol for which more than one stereospecific molecule exists. For the Acylcarnitine sub pathway: a capital C is followed by the number of carbons within the fatty acyl group attached to the carnitine. A colon followed by a number is one or more unsaturated carbons in the acylcarnitine ester (i.e., C10:1 is a monounsaturated C10 acylcarnitine). DC following the carbon number is a dicarboxylic acylcarnitine. Acylcarnitines are classified by the number of carbon atoms in the acyl group chain: short-chain acylcarnitines C2 to C7; medium-chain acylcarnitines C8 to C14; long-chain acylcarnitines C16 – C26 [30]. A summary of all 983 metabolites identified is present in Supplementary Data 2.

**Curation:** A variety of curation procedures were carried out to ensure that a high quality data set was made available for statistical analysis and data interpretation. The QC and curation processes were designed to ensure accurate and consistent identification of true chemical entities, and to remove those representing system artifacts, mis-assignments, and background noise. Metabolon data analysts use proprietary visualization and interpretation software to confirm the consistency of peak identification among the various samples. Library matches for each compound were checked for each sample and corrected if necessary.

**Metabolite Quantification and Data Normalization:** Peaks were quantified using total spectral area (area under the curve) [31-33]. Metabolite quantitation or abundance is defined as the total ion count for the given mass-to-charge ratio (*m/z*) assigned to the particular metabolite [34]. Specifically, metabolite quantitation is determined using extracted ion chromatograms by focusing the narrow mass window on the theoretical *m/z* value of the individual metabolite of interest and eliminating overlapping isobaric signals with maintenance of the mass accuracy during the acquisition [35-39]. A data normalization step was performed to correct variation resulting from instrument inter-day tuning differences. Each compound was corrected in run-day blocks by registering the medians to equal one (1.00) and normalizing each data point proportionately.

**mtDNA level Measurement:** In the RoCI cohort, cell-free plasma ND1 mtDNA levels were previously determined on 73 patients [10, 11]. Preparation and Quantification of plasma ND1 mtDNA have been outlined at length [10]. Briefly, in RoCI, blood samples were drawn and transferred into blood collection tubes within 24 hours from study inclusion and processed within 2 hours after venipuncture [40]. Plasma used in this study was prepared by using EDTA-coated blood collection tubes. The plasma samples stored at –80°C were thawed on ice. The 100 µl of plasma was mixed with 100 µl of PBS, followed by brief vortex. The diluted plasma was centrifuged at 700g at 4°C for 5 minutes and the supernatant (190 µl) was carefully saved by avoiding touching any pellets and the bottom of the tubes. The obtained supernatant was further centrifuged at 18,000g at 4°C for 15 minutes and the resulting supernatant (170 µl) was carefully saved and processed for DNA isolation using DNeasy Blood and Tissue (Qiagen; Part Number 69504) according to the manufacture manual [41]. For real-time quantitative polymerase chain reaction (qPCR) assay, the DNA solution was further diluted 10 times with nuclease-free deionized-distilled H_2_O.

DNA level in diluted samples was measured by SYBR® Green dye-based real-time qPCR assay PRISM 7300 sequence detection system (Applied Biosystems, Foster City, CA, USA). The references for primer sequences were as follows: Human NADH dehydrogenase 1 gene (ND1 mtDNA) [42, 43]; Human β-globin (nuclear DNA)[44]; Bacterial 16S ribosomal RNA [42]. Plasmid DNA with cDNA sequences for human ND1 mtDNA was obtained from ORIGENE (SC101172, Rockville, MD) and plasmid DNA with cDNA sequences for human nuclear DNA was obtained from Sino Biological Inc. Concentrations were converted to copy number using the formula; mol/g × molecules/mol = molecules/g, via a DNA copy number calculator http://www.uri.edu/research/gsc/resources/cndna.html [45, 46]. DNA solutions were diluted in 10-fold serial dilutions and used as standards.

All samples were analyzed in duplicate, and a no-template control was included in every analysis. ND1 mtDNA levels in all of the plasma analyses were expressed in copies per micro liter of plasma based on the following calculation [47]: c = Q X VDNA/VPCR X 1/Vext. c is the concentration of DNA in plasma (copies/µl plasma); Q is the quantity (copies) of DNA determined by the sequence detector in a PCR; VDNA is the total volume of plasma DNA solution obtained after extraction; typically 200 µl per extraction; VPCR is the volume of plasma DNA solution used for PCR, typically 5 µl of 10 times-diluted plasma DNA solution; Vext is the volume of plasma extracted, typically 50-100 µl [10].

**Statistical Analysis:** Determination of the changes in relative concentrations of metabolites was first suggested as a strategy to define the metabolome in 1998 [48]. Metabolomic profiling identified 983 metabolites (Supplementary Data 2). Metabolomic data underwent a cube root transformation followed by Pareto scaling to generate data that were on the same scale and followed an approximate normal distribution [49, 50].

The exposure of interest was N-formylmethionine abundance determined by liquid chromatography coupled mass spectrometry by Metabolon, Inc and analyzed as continuous. Elevated N-formylmethionine was defined as relative abundance in the top quartile relative to other patients within the same cohort (VITdAL-ICU or RoCI). The primary outcome was all cause mortality. Distributions of and differences in crude survival between quartiles of N-formylmethionine were determined the Kaplan*-*Meier survival curve and the log-rank test, respectively [51].

For univariate analysis of day 0 data, Student’s t-test was performed to determine if significant N-formylmethionine-specific differences exist using MetaboAnalyst [52]. We corrected for multiple testing via the Benjamini-Hochberg procedure to adjust the false discovery rate (FDR) to 0.05, producing a q-value which was used to identify all significant differences.[53] Day 0 data was also analyzed using orthogonal partial least square-discriminant analysis (OPLS-DA), also known as orthogonal projections to latent structures discriminant analysis, a supervised method to assess the significance of classification discrimination (SIMCA 15.0 Umetrics, Umea, Sweden). OPLS-DA was performed to relate the X data to the Y response [54, 55]. In our study, the X are the metabolites at day 0 and the Y is the exposure (N-formylmethionine). We assessed the OPLS-DA model quality via the variation of X explained by the model (R2X(cum)); the goodness-of-fit represented by the percentage of the variation of Y explained by the model (R2); and the predictive performance (Q2). Permutation testing was performed to validate the OPLS-DA model [56, 57]. The percentage of the variation of the dataset predicted by the model (Permuted Q2) was assessed using a cross-validation test [58, 59]. Sevenfold cross-validation analysis of variance (CV-ANOVA) was utilized to determine OPLS-DA model significance [57]. Additionally, response permutation testing was performed to validate the OPLS-DA model [56, 57]. To this end, the intervention is permutated to appear in a different order while the metabolite-dataset remains intact. Next, a model is then fit to the permutated data. The goodness-of-fit (R2) and predictive performance (Q2) of the permutated model are contrasted to the actual model. A valid model has lower permutated Q2 values compared to the actual model and the Q2-intercept is below zero [60].

For single time point data in the VITdAL-ICU cohort, correlations between individual metabolites and N-formylmethionine at day 0, 3 or 7 were separately determined utilizing linear regression models utilizing robust standard errors correcting for age, sex, SAPS II, admission diagnosis, and 25(OH)D at day 0. Additionally, for day 3, and 7, the linear regression models were corrected for absolute change in 25(OH)D level at day 3. A q-value of 0.05 was used for all significant associations [53]. We performed linear regression diagnostics to evaluate model assumptions including variance inflation factor, Breusch-Pagan / Cook-Weisberg test, normality of residuals and Cook's distance. We employed rain plots [61] to visualize effect size, significance, clustering and trends across days 0, 3, and 7. Rain plots were produced in R-3.6.2 adapted from source code published by Henglin et.al. [61].

For single time point data in the RoCI cohort, correlations between individual metabolites and N-formylmethionine at day 0 were determined utilizing linear regression models utilizing robust standard errors correcting for age, sex, race, and APACHE II score. A q-value of 0.05 was used for all significant associations [53]. All linear regression models were analyzed using STATA 16.1MP (College Station, TX).

To identify N-formylmethionine-specific modules from metabolomics data, we estimated Gaussian graphical models (GGMs) for day 3 and 7. Modules serve to reconstruct pathway reactions from metabolomics data. GGMs are determined utilizing partial pairwise Pearson correlation coefficients following the removal of the effects of all other metabolites and covariates [62]. GGMs are representations of the linear association between two metabolites corrected for other confounding variables in multivariate Gaussian distributions. We inferred a N-formylmethionine-specific network for relative metabolite abundance. We included age, sex, SAPS II, admission diagnosis, 25(OH)D at day 0, absolute change in 25(OH)D level at day 3, and plasma day as covariates into the model. Edges between metabolites were allotted if both their Pearson correlations and partial correlations remained statistically significant at P-value < 0.05 at a 5% FDR threshold [53]. GGMs were produced using the GeneNet R package, version 1.2.13 in R-3.6.2 adapted from source code published by Do et.al. [63].

**Supplementary Methods References Cited**

1. Amrein K, Schnedl C, Holl A, Riedl R, Christopher KB, Pachler C, Urbanic Purkart T, Waltensdorfer A, Munch A, Warnkross H *et al*: **Effect of high-dose vitamin D3 on hospital length of stay in critically ill patients with vitamin D deficiency: the VITdAL-ICU randomized clinical trial**. *JAMA* 2014, **312**(15):1520-1530.

2. World Medical A: **World Medical Association Declaration of Helsinki: ethical principles for medical research involving human subjects**. *JAMA* 2013, **310**(20):2191-2194.

3. Le Gall JR, Lemeshow S, Saulnier F: **A new Simplified Acute Physiology Score (SAPS II) based on a European/North American multicenter study**. *JAMA* 1993, **270**(24):2957-2963.

4. Dolinay T, Kim YS, Howrylak J, Hunninghake GM, An CH, Fredenburgh L, Massaro AF, Rogers A, Gazourian L, Nakahira K *et al*: **Inflammasome-regulated cytokines are critical mediators of acute lung injury**. *Am J Respir Crit Care Med* 2012, **185**(11):1225-1234.

5. Levy MM, Fink MP, Marshall JC, Abraham E, Angus D, Cook D, Cohen J, Opal SM, Vincent JL, Ramsay G: **2001 SCCM/ESICM/ACCP/ATS/SIS International Sepsis Definitions Conference**. *Crit Care Med* 2003, **31**(4):1250-1256.

6. Bernard GR, Artigas A, Brigham KL, Carlet J, Falke K, Hudson L, Lamy M, Legall JR, Morris A, Spragg R: **The American-European Consensus Conference on ARDS. Definitions, mechanisms, relevant outcomes, and clinical trial coordination**. *Am J Respir Crit Care Med* 1994, **149**(3 Pt 1):818-824.

7. Sohn MW, Arnold N, Maynard C, Hynes DM: **Accuracy and completeness of mortality data in the Department of Veterans Affairs**. *Popul Health Metr* 2006, **4**:2.

8. Knaus WA, Draper EA, Wagner DP, Zimmerman JE: **APACHE II: a severity of disease classification system**. *Crit Care Med* 1985, **13**(10):818-829.

9. Rogers AJ, McGeachie M, Baron RM, Gazourian L, Haspel JA, Nakahira K, Fredenburgh LE, Hunninghake GM, Raby BA, Matthay MA *et al*: **Metabolomic derangements are associated with mortality in critically ill adult patients**. *PLoS One* 2014, **9**(1):e87538.

10. Nakahira K, Kyung SY, Rogers AJ, Gazourian L, Youn S, Massaro AF, Quintana C, Osorio JC, Wang Z, Zhao Y *et al*: **Circulating mitochondrial DNA in patients in the ICU as a marker of mortality: derivation and validation**. *PLoS medicine* 2013, **10**(12):e1001577.

11. Johansson PI, Nakahira K, Rogers AJ, McGeachie MJ, Baron RM, Fredenburgh LE, Harrington J, Choi AMK, Christopher KB: **Plasma mitochondrial DNA and metabolomic alterations in severe critical illness**. *Crit Care* 2018, **22**(1):360.

12. Mogensen KM, Lasky-Su J, Rogers AJ, Baron RM, Fredenburgh LE, Rawn J, Robinson MK, Massarro A, Choi AM, Christopher KB: **Metabolites Associated With Malnutrition in the Intensive Care Unit Are Also Associated With 28-Day Mortality**. *JPEN J Parenter Enteral Nutr* 2017, **41**(2):188-197.

13. Lasky-Su J, Dahlin A, Litonjua AA, Rogers AJ, McGeachie MJ, Baron RM, Gazourian L, Barragan-Bradford D, Fredenburgh LE, Choi AMK *et al*: **Metabolome alterations in severe critical illness and vitamin D status**. *Crit Care* 2017, **21**(1):193.

14. Breier M, Wahl S, Prehn C, Fugmann M, Ferrari U, Weise M, Banning F, Seissler J, Grallert H, Adamski J *et al*: **Targeted metabolomics identifies reliable and stable metabolites in human serum and plasma samples**. *PLoS One* 2014, **9**(2):e89728.

15. Chary S, Amrein K, Lasky-Su J, Dobnig H, Christopher KB: **The Sex-specific Metabolic Response to Critical Illness: a post-hoc metabolomics study of the VITdAL-ICU trial**. *Scientific Reports* 2021, **11**:3951.

16. Amrein K, Lasky-Su JA, Dobnig H, Christopher KB: **Metabolomic basis for response to high dose vitamin D in critical illness**. *Clinical nutrition* 2020.

17. Wehrens R, Hageman JA, van Eeuwijk F, Kooke R, Flood PJ, Wijnker E, Keurentjes JJ, Lommen A, van Eekelen HD, Hall RD *et al*: **Improved batch correction in untargeted MS-based metabolomics**. *Metabolomics* 2016, **12**:88.

18. Trezzi JP, Jager C, Galozzi S, Barkovits K, Marcus K, Mollenhauer B, Hiller K: **Metabolic profiling of body fluids and multivariate data analysis**. *MethodsX* 2017, **4**:95-103.

19. Bain JR, Stevens RD, Wenner BR, Ilkayeva O, Muoio DM, Newgard CB: **Metabolomics applied to diabetes research: moving from information to knowledge**. *Diabetes* 2009, **58**(11):2429-2443.

20. Parsons HM, Ekman DR, Collette TW, Viant MR: **Spectral relative standard deviation: a practical benchmark in metabolomics**. *Analyst* 2009, **134**(3):478-485.

21. Narvaez-Rivas M, Zhang Q: **Comprehensive untargeted lipidomic analysis using core-shell C30 particle column and high field orbitrap mass spectrometer**. *J Chromatogr A* 2016, **1440**:123-134.

22. Michopoulos F, Lai L, Gika H, Theodoridis G, Wilson I: **UPLC-MS-based analysis of human plasma for metabonomics using solvent precipitation or solid phase extraction**. *J Proteome Res* 2009, **8**(4):2114-2121.

23. Want EJ, Smith CA, Qin C, Van Horne KC, Siuzdak G: **Phospholipid capture combined with non-linear chromatographic correction for improved serum metabolite profiling**. *Metabolomics* 2006, **2**:145-154.

24. Oresic M, Vidal-Puig A, Hanninen V: **Metabolomic approaches to phenotype characterization and applications to complex diseases**. *Expert Rev Mol Diagn* 2006, **6**(4):575-585.

25. Chen WW, Freinkman E, Wang T, Birsoy K, Sabatini DM: **Absolute Quantification of Matrix Metabolites Reveals the Dynamics of Mitochondrial Metabolism**. *Cell* 2016, **166**(5):1324-1337 e1311.

26. Hufsky F, Scheubert K, Bocker S: **Computational mass spectrometry for small-molecule fragmentation**. *TrAC Trends Anal Chem* 2014, **53**:41–48.

27. Dunn WB, Broadhurst D, Begley P, Zelena E, Francis-McIntyre S, Anderson N, Brown M, Knowles JD, Halsall A, Haselden JN *et al*: **Procedures for large-scale metabolic profiling of serum and plasma using gas chromatography and liquid chromatography coupled to mass spectrometry**. *Nat Protoc* 2011, **6**(7):1060-1083.

28. Sumner LW, Amberg A, Barrett D, Beale MH, Beger R, Daykin CA, Fan TW, Fiehn O, Goodacre R, Griffin JL *et al*: **Proposed minimum reporting standards for chemical analysis Chemical Analysis Working Group (CAWG) Metabolomics Standards Initiative (MSI)**. *Metabolomics* 2007, **3**(3):211-221.

29. Schrimpe-Rutledge AC, Codreanu SG, Sherrod SD, McLean JA: **Untargeted Metabolomics Strategies-Challenges and Emerging Directions**. *J Am Soc Mass Spectrom* 2016, **27**(12):1897-1905.

30. Guasch-Ferre M, Zheng Y, Ruiz-Canela M, Hruby A, Martinez-Gonzalez MA, Clish CB, Corella D, Estruch R, Ros E, Fito M *et al*: **Plasma acylcarnitines and risk of cardiovascular disease: effect of Mediterranean diet interventions**. *The American journal of clinical nutrition* 2016, **103**(6):1408-1416.

31. Weljie AM, Newton J, Mercier P, Carlson E, Slupsky CM: **Targeted profiling: quantitative analysis of 1H NMR metabolomics data**. *Anal Chem* 2006, **78**(13):4430-4442.

32. Wishart DS: **Quantitative metabolomics using NMR**. *TrAC Trends Anal Chem* 2008, **27**:228–237.

33. Zhou B, Xiao JF, Tuli L, Ressom HW: **LC-MS-based metabolomics**. *Mol Biosyst* 2012, **8**(2):470-481.

34. Parisi LR, Li N, Atilla-Gokcumen GE: **Very Long Chain Fatty Acids Are Functionally Involved in Necroptosis**. *Cell Chem Biol* 2017, **24**(12):1445-1454 e1448.

35. Junot C, Madalinski G, Tabet JC, Ezan E: **Fourier transform mass spectrometry for metabolome analysis**. *Analyst* 2010, **135**(9):2203-2219.

36. Kamleh A, Barrett MP, Wildridge D, Burchmore RJ, Scheltema RA, Watson DG: **Metabolomic profiling using Orbitrap Fourier transform mass spectrometry with hydrophilic interaction chromatography: a method with wide applicability to analysis of biomolecules**. *Rapid Commun Mass Spectrom* 2008, **22**(12):1912-1918.

37. Kamleh MA, Hobani Y, Dow JA, Watson DG: **Metabolomic profiling of Drosophila using liquid chromatography Fourier transform mass spectrometry**. *FEBS Lett* 2008, **582**(19):2916-2922.

38. Koulman A, Woffendin G, Narayana VK, Welchman H, Crone C, Volmer DA: **High-resolution extracted ion chromatography, a new tool for metabolomics and lipidomics using a second-generation orbitrap mass spectrometer**. *Rapid Commun Mass Spectrom* 2009, **23**(10):1411-1418.

39. Xiao JF, Zhou B, Ressom HW: **Metabolite identification and quantitation in LC-MS/MS-based metabolomics**. *Trends Analyt Chem* 2012, **32**:1-14.

40. Dolinay T, Kim YS, Howrylak J, Hunninghake GM, An CH, Fredenburgh L, Massaro AF, Rogers A, Gazourian L, Nakahira K *et al*: **Inflammasome-Regulated Cytokines are Critical Mediators of Acute Lung Injury**. *Am J Respir Crit Care Med* 2012.

41. Nakahira K, Haspel JA, Rathinam VA, Lee SJ, Dolinay T, Lam HC, Englert JA, Rabinovitch M, Cernadas M, Kim HP *et al*: **Autophagy proteins regulate innate immune responses by inhibiting the release of mitochondrial DNA mediated by the NALP3 inflammasome**. *Nat Immunol* 2011, **12**(3):222-230.

42. Zhang Q, Raoof M, Chen Y, Sumi Y, Sursal T, Junger W, Brohi K, Itagaki K, Hauser CJ: **Circulating mitochondrial DAMPs cause inflammatory responses to injury**. *Nature* 2010, **464**(7285):104-107.

43. McGill MR, Sharpe MR, Williams CD, Taha M, Curry SC, Jaeschke H: **The mechanism underlying acetaminophen-induced hepatotoxicity in humans and mice involves mitochondrial damage and nuclear DNA fragmentation**. *The Journal of clinical investigation* 2012, **122**(4):1574-1583.

44. Moreira VG, Prieto B, Rodriguez JS, Alvarez FV: **Usefulness of cell-free plasma DNA, procalcitonin and C-reactive protein as markers of infection in febrile patients**. *Annals of clinical biochemistry* 2010, **47**(Pt 3):253-258.

45. Nga TV, Karkey A, Dongol S, Thuy HN, Dunstan S, Holt K, Tu le TP, Campbell JI, Chau TT, Chau NV *et al*: **The sensitivity of real-time PCR amplification targeting invasive Salmonella serovars in biological specimens**. *BMC infectious diseases* 2010, **10**:125.

46. Zozaya-Hinchliffe M, Martin DH, Ferris MJ: **Prevalence and abundance of uncultivated Megasphaera-like bacteria in the human vaginal environment**. *Applied and environmental microbiology* 2008, **74**(5):1656-1659.

47. Chiu RW, Chan LY, Lam NY, Tsui NB, Ng EK, Rainer TH, Lo YM: **Quantitative analysis of circulating mitochondrial DNA in plasma**. *Clinical chemistry* 2003, **49**(5):719-726.

48. Oliver SG, Winson MK, Kell DB, Baganz F: **Systematic functional analysis of the yeast genome**. *Trends Biotechnol* 1998, **16**(9):373-378.

49. van den Berg RA, Hoefsloot HC, Westerhuis JA, Smilde AK, van der Werf MJ: **Centering, scaling, and transformations: improving the biological information content of metabolomics data**. *BMC Genomics* 2006, **7**:142.

50. Struja T, Eckart A, Kutz A, Huber A, Neyer P, Kraenzlin M, Mueller B, Meier C, Bernasconi L, Schuetz P: **Metabolomics for Prediction of Relapse in Graves' Disease: Observational Pilot Study**. *Front Endocrinol (Lausanne)* 2018, **9**:623.

51. Kaplan E, Meier P: **Nonparametric estimation from incomplete observations**. *J AM Stat Assn* 1958, **53**:457-481.

52. Chong J, Xia J: **Using MetaboAnalyst 4.0 for Metabolomics Data Analysis, Interpretation, and Integration with Other Omics Data**. *Methods Mol Biol* 2020, **2104**:337-360.

53. Benjamini Y, Hochberg Y: **Controlling for false discovery rate: a practical and powerful approach to multiple testing.** *Journal of the Royal Statistical Society Series B (Methodological)* 1995, **57**:289–300.

54. Bylesjö M, Rantalainen M, Cloarec O, Nicholson JK, Holmes E, Trygg J: **OPLS discriminant analysis: combining the strengths of PLS-DA and SIMCA classification**. *Journal of Chemometrics* 2006, **20**(8-10):341–351.

55. Trygg J, Wold S: **Orthogonal projections to latent structures (O-PLS)**. *J Chemometrics* 2002, **16**:119–128.

56. Westerhuis JA, Hoefsloot HCJ, Smit S, Vis DJ, Smilde AK, van Velzen EJJ, van Duijnhoven JPM, van Dorsten FA: **Assessment of PLSDA cross validation**. *Metabolomics* 2008, **4**(1):81–89.

57. Eriksson L, Trygg J, Wold S: **CV‐ANOVA for significance testing of PLS and OPLS models**. *Journal of Chemometrics* 2008, **22**(11-12):594-600.

58. Eastment H, Krzanowski W: **Crossvalidatory choice of the number of components from a principal component analysis**. *Technometrics* 1982, **24**:73-77.

59. Martens H, Naes T: **Multivariate Calibration**. Chichester: John Wiley and Sons; 1989.

60. Mahadevan S, Shah SL, T.J. M, Slupsky CM: **Analysis of Metabolomic Data Using Support Vector Machines**. *Analytical Chemistry* 2008, **80**(19):7562–7570.

61. Henglin M, Niiranen T, Watrous JD, Lagerborg KA, Antonelli J, Claggett BL, Demosthenes EJ, von Jeinsen B, Demler O, Vasan RS *et al*: **A Single Visualization Technique for Displaying Multiple Metabolite-Phenotype Associations**. *Metabolites* 2019, **9**(7).

62. Krumsiek J, Suhre K, Illig T, Adamski J, Theis FJ: **Gaussian graphical modeling reconstructs pathway reactions from high-throughput metabolomics data**. *BMC Syst Biol* 2011, **5**:21.

63. Do KT, Pietzner M, Rasp DJ, Friedrich N, Nauck M, Kocher T, Suhre K, Mook-Kanamori DO, Kastenmuller G, Krumsiek J: **Phenotype-driven identification of modules in a hierarchical map of multifluid metabolic correlations**. *NPJ Syst Biol Appl* 2017, **3**:28.
