# Supplementary material for: Circulating N-formylmethionine and metabolic shift in critical illness: a multicohort metabolomics study
Source: Crit Care. 2022 Oct 19;26:321. doi: 10.1186/s13054-022-04174-y (PMC9580206; doi:10.1186/s13054-022-04174-y)
Supplement: Supplementary file 2 — Additional file 2. Admission Diagnosis category of VITdAL-ICU cohort patients by day 0 N-Formylmethionine abundance quartiles. [file 13054_2022_4174_MOESM2_ESM.docx]

**Additional file 2: Admission Diagnosis category of VITdAL-ICU cohort patients by day 0 N-Formylmethionine abundance quartiles**

| **Characteristics** | **N-Formylmethionine Abundance at day 0** | | | |
| --- | --- | --- | --- | --- |
|  | **Q1** | **Q2** | **Q3** | **Q4** |
| **VITdAL-ICU cohort** |  |  |  |  |
| No. | 107 | 107 | 107 | 107 |
| Neurosurgery No. (%) | 2 (2) | 1 (1) | 1 (1) | 0 (0) |
| Cardiac surgery No. (%) | 11 (10) | 19 (18) | 25 (23) | 26 (24) |
| Cardiovascular No. (%) | 16 (15) | 8 (7) | 16 (15) | 10 (9) |
| Gastrointestinal/liver No. (%) | 5 (5) | 0 (0) | 4 (4) | 4 (4) |
| Hematologic/oncologic No. (%) | 1 (1) | 0 (0) | 0 (0) | 0 (0) |
| Metabolic/Renal No. (%) | 0 (0) | 1 (1) | 1 (1) | 1 (1) |
| Neurologic No. (%) | 34 (32) | 34 (32) | 19 (18) | 17 (16) |
| Other non-operative No. (%) | 1 (1) | 1 (1) | 1 (1) | 0 (0) |
| Other operative No. (%) | 3 (3) | 2 (2) | 2 (2) | 5 (5) |
| Respiratory No. (%) | 0 (0) | 0 (0) | 1 (1) | 3 (3) |
| Sepsis No. (%) | 6 (6) | 7 (7) | 10 (9) | 13 (12) |
| Infectious No. (%) | 2 (2) | 9 (8) | 7 (7) | 13 (12) |
| Thoracic surgery No. (%) | 4 (4) | 4 (4) | 4 (4) | 2 (2) |
| Transplantation No. (%) | 1 (1) | 3 (3) | 1 (1) | 8 (7) |
| Trauma No. (%) | 4 (4) | 3 (3) | 4 (4) | 0 (0) |
| Vascular No. (%) | 16 (15) | 12 (11) | 6 (6) | 4 (4) |

Note: Data presented as number (No.) percentage (%).
