# Supplementary material for: Circulating N-formylmethionine and metabolic shift in critical illness: a multicohort metabolomics study
Source: Crit Care. 2022 Oct 19;26:321. doi: 10.1186/s13054-022-04174-y (PMC9580206; doi:10.1186/s13054-022-04174-y)
Supplement: Supplementary file 3 — Additional file 3. Associations between plasma N-formylmethionine abundance quartiles and 28-day mortality. Logistic regression models shown for crude and adjusted VITdAL-ICU and RoCI cohort survival data. Additional models shown with adjustment for total bilirubin, creatinine, and/or propofol exposure. [file 13054_2022_4174_MOESM3_ESM.docx]

**Additional file 3. Associations between plasma N-formylmethionine abundance quartiles and 28-day mortality**

| **Model** | **Odds Ratio** | **95% Confidence Interval** | **p-value** | **AIC** | **BIC** | **Likelihood-ratio test p-value** |
| --- | --- | --- | --- | --- | --- | --- |
| **VITdAL-ICU Cohort** |  |  |  |  |  |  |
| Unadjusted | 3.07 | 1.89, 5.00 | <0.001 |  |  |  |
| Adjusted |  |  |  |  |  |  |
| Model 1 | 2.42 | 1.45, 4.04 | 0.001 | 420 | 449 |  |
| Model 2 | 2.37 | 1.43, 3.93 | 0.001 | 424 | 449 | 0.017* |
| Model 3 | 2.21 | 1.30, 3.75 | 0.003 | 410 | 442 | <0.001* |
| Model 4 | 1.94 | 1.11, 3.39 | <0.001 | 419 | 447 | <0.001* |
| Model 5 | 2.57 | 1.52, 4.35 | <0.001 | 413 | 446 | 0.002* |
| Model 6 | 1.93 | 1.08, 3.45 | 0.027 | 406 | 442 | <0.001* |
| Model 7 | 1.93 | 1.09, 3.42 | 0.025 | 415 | 447 | 0.006* |
| **RoCI Cohort** |  |  |  |  |  |  |
| Unadjusted | 4.71 | 1.51, 14.69 | 0.008 |  |  |  |
| Adjusted |  |  |  |  |  |  |
| Model 8 | 5.06 | 1.37, 18.72 | 0.015 | 81 | 95 |  |
| Model 9 | 5.93 | 1.66, 21.15 | 0.006 | 81 | 92 | 0.24^†^ |
| Model 10 | 5.19 | 1.33, 20.29 | 0.018 | 76 | 91 | 0.005^†^ |
| Model 11 | 9.83 | 2.04, 47.24 | 0.004 | 77 | 92 | 0.01^†^ |
| Model 12 | 11.22 | 1.96, 64.29 | 0.007 | 71 | 89 | <0.001^†^ |
| Model 13 | 10.28 | 2.09, 50.48 | 0.004 | 73 | 89 | 00015^†^ |

Logistic regression models shown for crude and adjusted **VITdAL-ICU** and **RoCI** cohort survival data. Additional models shown with adjustment for total bilirubin, creatinine, **and/or** propofol exposure.

Note: Significant associations between plasma N-formylmethionine day 0 abundance quartiles and 28-day mortality were determined utilizing logistic regression.

In the VITdAL-ICU cohort (N=428), the unadjusted association was determined between 28-day mortality and the top quartile of plasma N-formylmethionine abundance at day 0.

Model 1 was adjusted for age, sex, baseline 25(OH)D, SAPS II, and admission diagnosis.

Model 2 was adjusted for age, sex, baseline 25(OH)D, and admission diagnosis.

Model 3 was adjusted for age, sex, baseline 25(OH)D, SAPS II, admission diagnosis, and total bilirubin at day 0.

Model 4 was adjusted for age, sex, baseline 25(OH)D, SAPS II, admission diagnosis, and creatinine at day 0.

Model 5 was adjusted for age, sex, baseline 25(OH)D, SAPS II, admission diagnosis, and propofol exposure at day 0.

Model 6 was adjusted for age, sex, baseline 25(OH)D, SAPS II, admission diagnosis, total bilirubin at day 0, creatinine at day 0, and propofol exposure at day 0.

Model 7 was adjusted for age, sex, baseline 25(OH)D, admission diagnosis, total bilirubin at day 0, creatinine at day 0, and propofol exposure at day 0.

In the RoCI cohort (N=90), the unadjusted association was determined between 28-day mortality and the top quartile of plasma N-formylmethionine abundance at day 0.

Model 8 was adjusted for age, sex, race, and APACHE II score.

Model 9 was adjusted for age, sex, and race.

Model 10 was adjusted for age, sex, race, APACHE II score, and total bilirubin at day 0.

Model 11 was adjusted for age, sex, race, APACHE II score, and creatinine at day 0.

Model 12 was adjusted for age, sex, race, APACHE II score, total bilirubin at day 0, and creatinine at day 0.

Model 13 was adjusted for was adjusted for age, sex, race, total bilirubin at day 0, and creatinine at day 0.

* Likelihood-ratio test comparing Model 1 to Model 2, 3, 4, 5, 6, or 7.

^†^ Likelihood-ratio test comparing Model 8 to Model 9, 10, 11, 12, or 13.
