# Supplementary material for: Circulating N-formylmethionine and metabolic shift in critical illness: a multicohort metabolomics study
Source: Crit Care. 2022 Oct 19;26:321. doi: 10.1186/s13054-022-04174-y (PMC9580206; doi:10.1186/s13054-022-04174-y)
Supplement: Supplementary file 5 — Additional file 5. Dot Boxplots of metabolite Sub Pathways by N-formylmethionine quartiles at Day 0. Unadjusted normalized abundance of metabolites from 428 VITdAL-ICU cohort subjects at day 0 by quartiles of N-formylmethionine abundance. Sub Pathways shown include short-chain acylcarnitine, branched chain amino acid, pentose phosphate pathway and purine metabolites. Box plots show the data between the first and third quartile (box), the median and whiskers at 1.5 times the interquartile range. All individual data points are visualized using a bee swarm plot. [file 13054_2022_4174_MOESM5_ESM.pptx]

## Slide 1
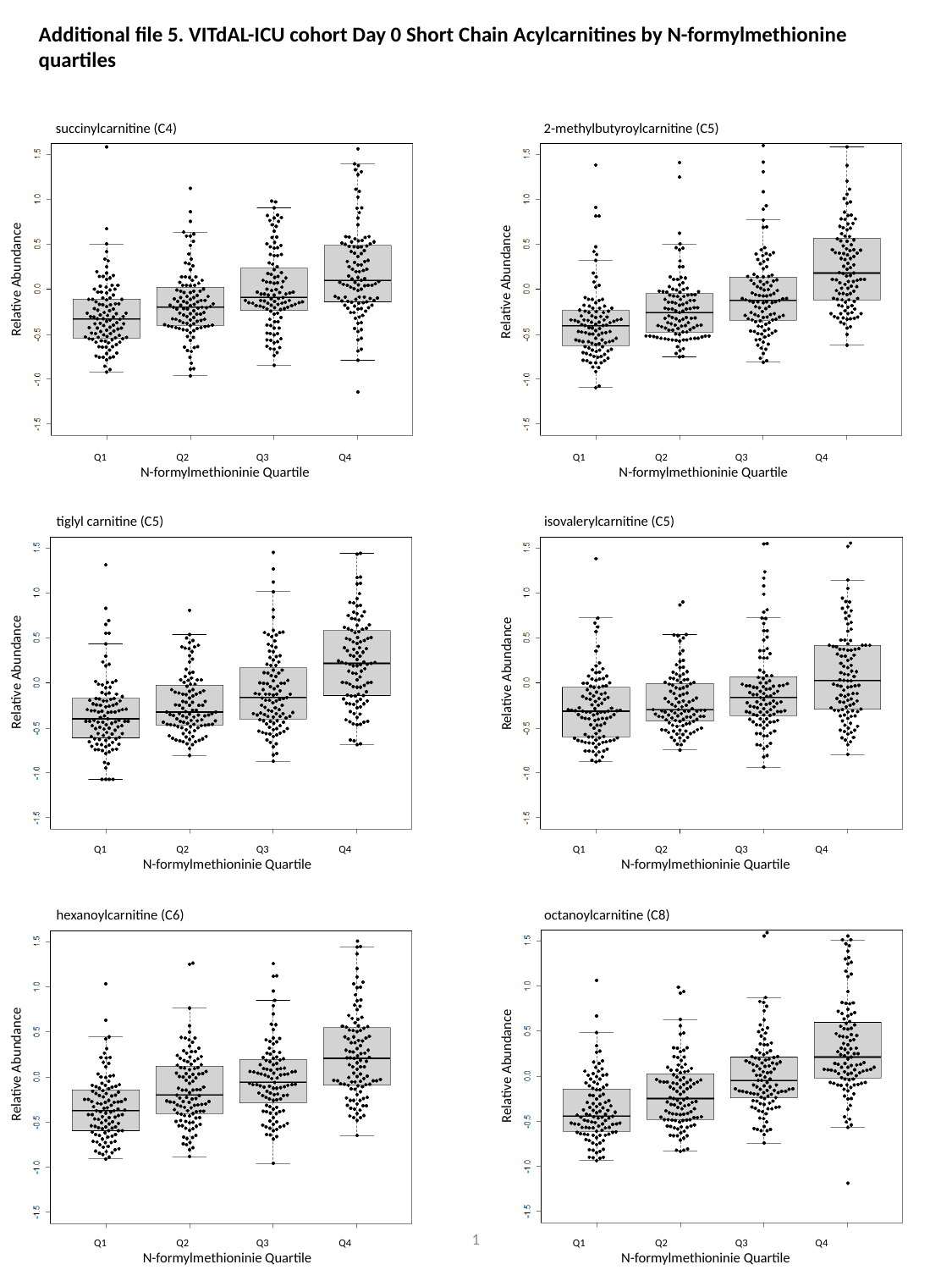

Additional file 5. VITdAL-ICU cohort Day 0 Short Chain Acylcarnitines by N-formylmethionine quartiles
succinylcarnitine (C4)
2-methylbutyroylcarnitine (C5)
Relative Abundance
Relative Abundance
 Q1 Q2 Q3 Q4 Q1 Q2 Q3 Q4
 N-formylmethioninie Quartile N-formylmethioninie Quartile
tiglyl carnitine (C5)
isovalerylcarnitine (C5)
Relative Abundance
Relative Abundance
 Q1 Q2 Q3 Q4 Q1 Q2 Q3 Q4
 N-formylmethioninie Quartile N-formylmethioninie Quartile
hexanoylcarnitine (C6)
octanoylcarnitine (C8)
Relative Abundance
Relative Abundance
1
 Q1 Q2 Q3 Q4 Q1 Q2 Q3 Q4
 N-formylmethioninie Quartile N-formylmethioninie Quartile

## Slide 2
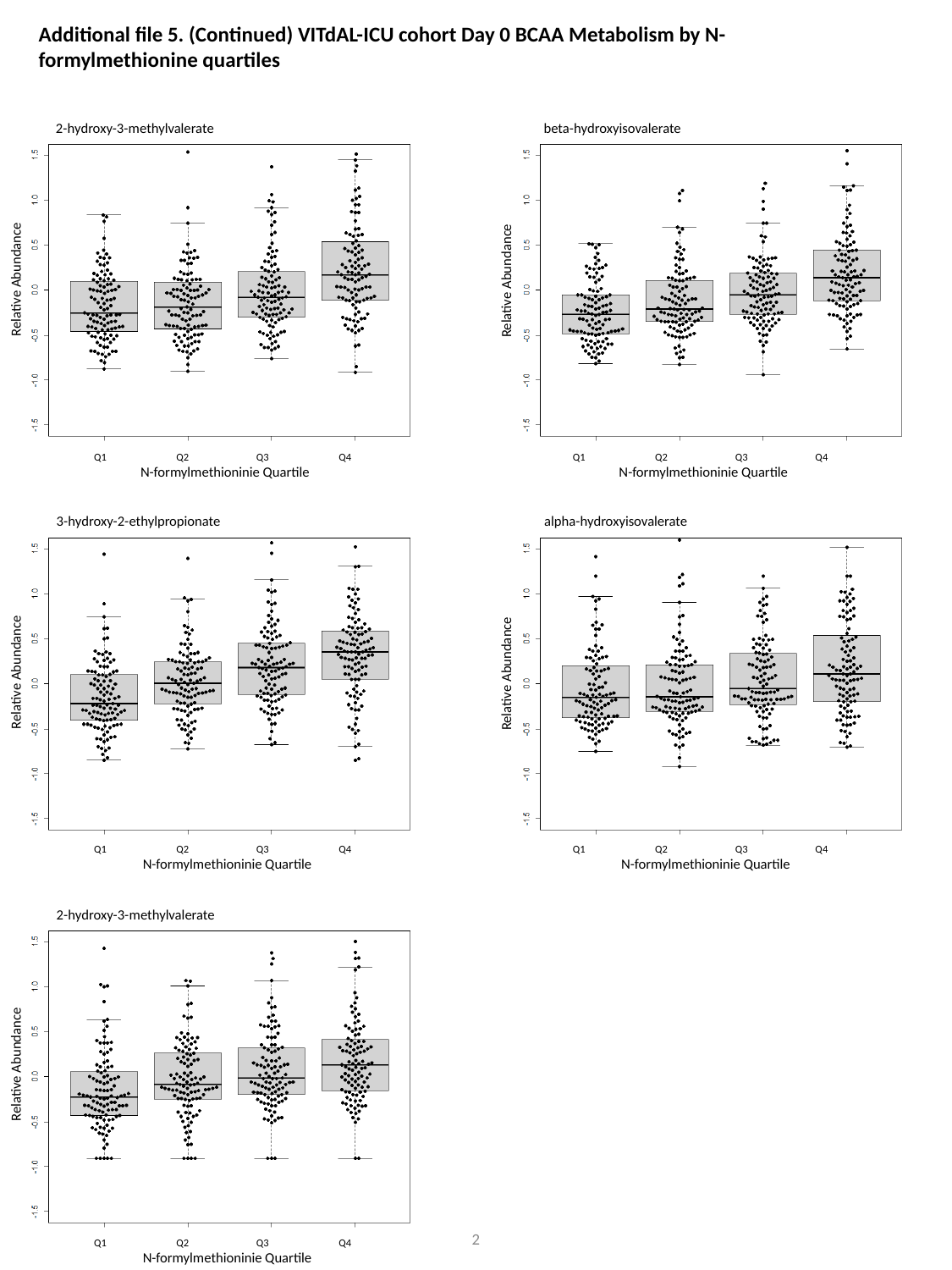

Additional file 5. (Continued) VITdAL-ICU cohort Day 0 BCAA Metabolism by N-formylmethionine quartiles
2-hydroxy-3-methylvalerate
beta-hydroxyisovalerate
Relative Abundance
Relative Abundance
Relative Abundance
 Q1 Q2 Q3 Q4 Q1 Q2 Q3 Q4
 N-formylmethioninie Quartile N-formylmethioninie Quartile
3-hydroxy-2-ethylpropionate
alpha-hydroxyisovalerate
Relative Abundance
Relative Abundance
 Q1 Q2 Q3 Q4 Q1 Q2 Q3 Q4
 N-formylmethioninie Quartile N-formylmethioninie Quartile
2-hydroxy-3-methylvalerate
Relative Abundance
2
 Q1 Q2 Q3 Q4 Q1 Q2 Q3 Q4
 N-formylmethioninie Quartile N-formylmethioninie Quartile

## Slide 3
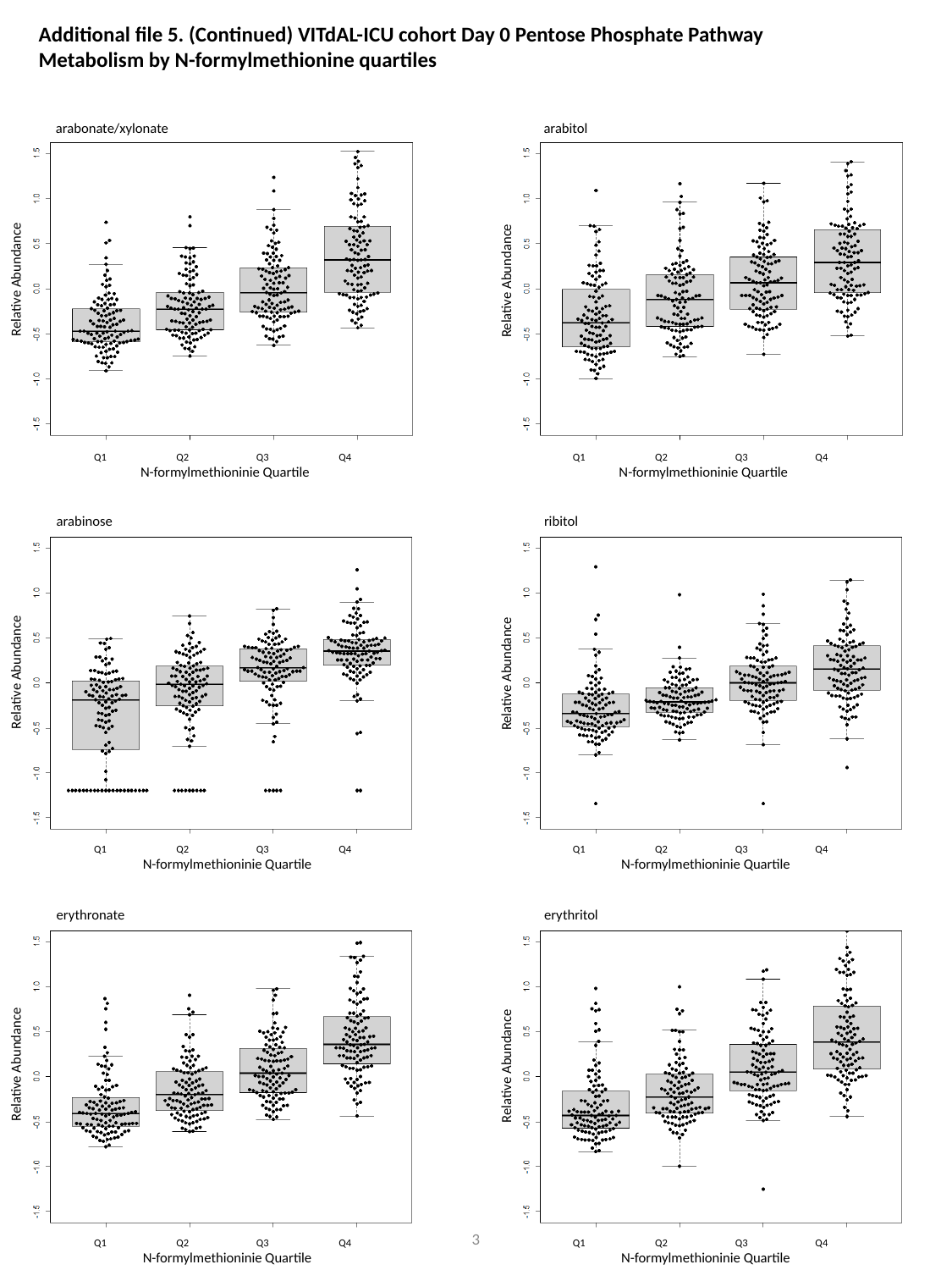

Additional file 5. (Continued) VITdAL-ICU cohort Day 0 Pentose Phosphate Pathway Metabolism by N-formylmethionine quartiles
arabonate/xylonate
arabitol
Relative Abundance
Relative Abundance
Relative Abundance
 Q1 Q2 Q3 Q4 Q1 Q2 Q3 Q4
 N-formylmethioninie Quartile N-formylmethioninie Quartile
arabinose
ribitol
Relative Abundance
Relative Abundance
 Q1 Q2 Q3 Q4 Q1 Q2 Q3 Q4
 N-formylmethioninie Quartile N-formylmethioninie Quartile
erythronate
erythritol
Relative Abundance
Relative Abundance
3
 Q1 Q2 Q3 Q4 Q1 Q2 Q3 Q4
 N-formylmethioninie Quartile N-formylmethioninie Quartile

## Slide 4
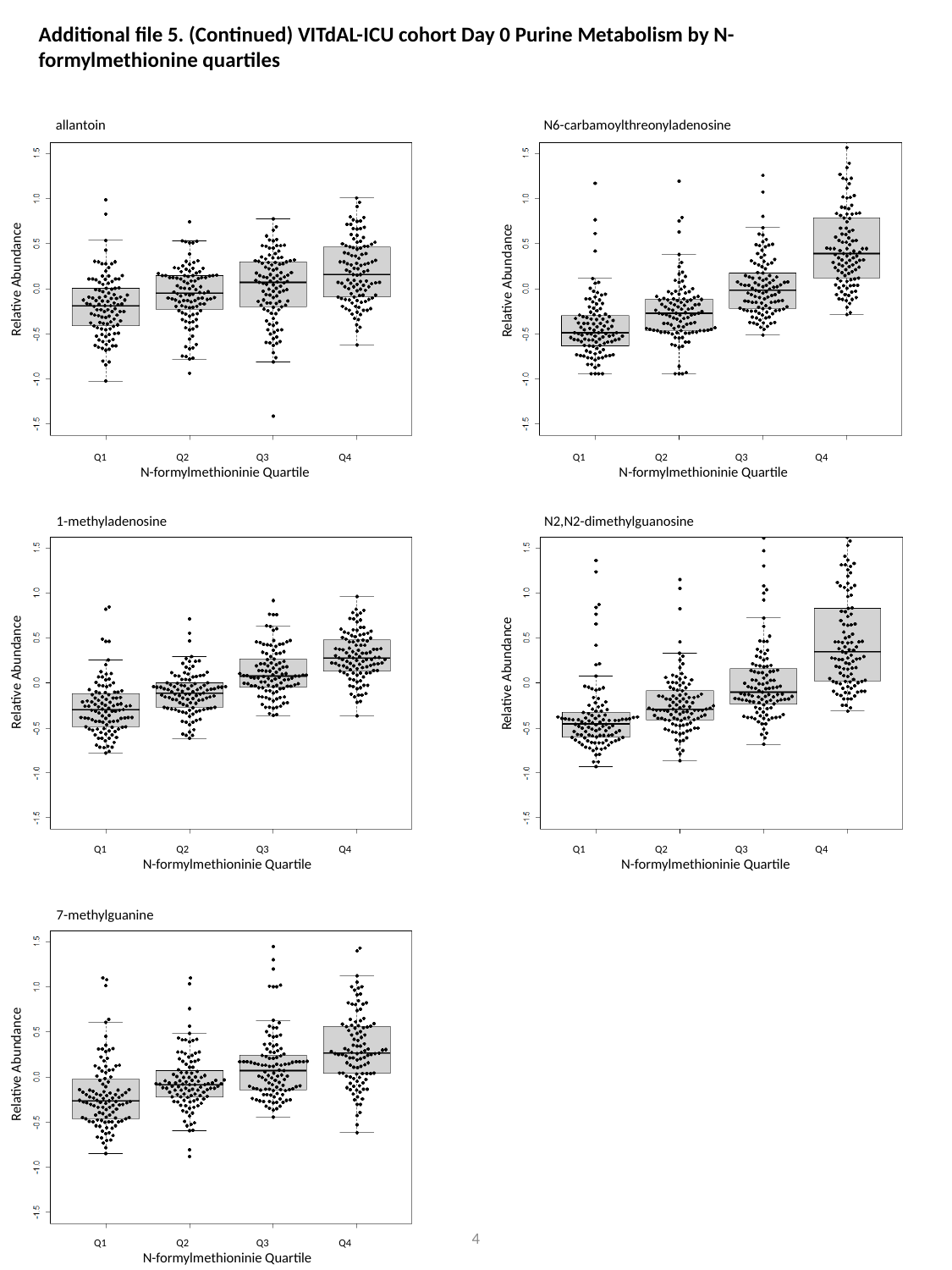

Additional file 5. (Continued) VITdAL-ICU cohort Day 0 Purine Metabolism by N-formylmethionine quartiles
allantoin
N6-carbamoylthreonyladenosine
Relative Abundance
Relative Abundance
 Q1 Q2 Q3 Q4 Q1 Q2 Q3 Q4
 N-formylmethioninie Quartile N-formylmethioninie Quartile
1-methyladenosine
N2,N2-dimethylguanosine
Relative Abundance
Relative Abundance
 Q1 Q2 Q3 Q4 Q1 Q2 Q3 Q4
 N-formylmethioninie Quartile N-formylmethioninie Quartile
7-methylguanine
Relative Abundance
4
 Q1 Q2 Q3 Q4 Q1 Q2 Q3 Q4
 N-formylmethioninie Quartile N-formylmethioninie Quartile
