# Supplementary material for: Circulating N-formylmethionine and metabolic shift in critical illness: a multicohort metabolomics study
Source: Crit Care. 2022 Oct 19;26:321. doi: 10.1186/s13054-022-04174-y (PMC9580206; doi:10.1186/s13054-022-04174-y)
Supplement: Supplementary file 6 — Additional file 6. Day 0 OPLS-DA model goodness of fit, predictive ability and model significance in both VITdAL-ICU and RoCI Cohorts. R2 and Q2 quality metrics, the permutation diagnostics and overall model significance are presented. [file 13054_2022_4174_MOESM6_ESM.docx]

**Additional file 6. Day 0 OPLS-DA model goodness of fit, predictive ability and model significance**

|  | **OPLS-DA** | | | **Permutation (n = 200)** | | **CV-ANOVA** |
| --- | --- | --- | --- | --- | --- | --- |
| Classification Model | R2X | R2Y | Q2 | R2 intercept  (x-axis, y-axis) | Q2 intercept  (x-axis, y-axis) | p-value |
| VITdAL-ICU Cohort | 0.126 | 1.00 | 0.350 | 0.00, 0.077 | 0.00, -0.102 | <0.001 |
| RoCI Cohort | 0.183 | 1.00 | 0.350 | 0.00, 0.274 | 0.00, -0.271 | <0.001 |

**Day 0 OPLS-DA model goodness of fit, predictive ability and model significance in both VITdAL-ICU and RoCI Cohorts.** R2 and Q2 quality metrics, the permutation diagnostics and overall model significance are presented.

Note: OPLS-DA Models are N-formylmethionine abundance 4^th^ quartile relative to 1^st^, 2^nd^ and 3^rd^ quartile. In our study, the X are the metabolites at day 0 and the Y is the N-formylmethionine abundance. The OPLS-DA model quality is R2X; the goodness-of-fit is R2Y; and the predictive performance is Q2. Model validation is determined via permutation testing and assessing the y-axis intercept Q2 by a cross-validation test. Sevenfold cross-validation analysis of variance (CV-ANOVA) was utilized for model significance. Further detail can be found in Supplemental Methods (Additional file 1).
