# Supplementary material for: Circulating N-formylmethionine and metabolic shift in critical illness: a multicohort metabolomics study
Source: Crit Care. 2022 Oct 19;26:321. doi: 10.1186/s13054-022-04174-y (PMC9580206; doi:10.1186/s13054-022-04174-y)
Supplement: Supplementary file 7 — Additional file 7. Highlighted Significantly Different Metabolites with increased N-formylmethionine abundance in the VITdAL-ICU Cohort at day 0. [file 13054_2022_4174_MOESM7_ESM.docx]

**Additional file 7. Highlighted Significantly Different Metabolites with increased N-formylmethionine abundance in the VITdAL-ICU Cohort at day 0**

| **Metabolite** | **Beta Coefficient** | **p-value** | **q-value** | **Super Pathway** | **Sub Pathway** |
| --- | --- | --- | --- | --- | --- |
| Lactate | 0.11 | 2.91 E-02 | 5.46 E-02 | Carbohydrate | Glycolysis, Gluconeogenesis, and Pyruvate Metabolism |
| 2,3-dihydroxy-2-methylbutyrate | 0.46 | 9.42 E-09 | 5.93 E-08 | Amino Acid | BCAA Metabolism |
| 2-hydroxy-3-methylvalerate | 0.40 | 8.69 E-06 | 3.16 E-05 | Amino Acid | BCAA Metabolism |
| 3-hydroxy-2-ethylpropionate | 0.33 | 1.50 E-07 | 7.36 E-07 | Amino Acid | BCAA Metabolism |
| 3-hydroxyisobutyrate | 0.27 | 1.78 E-03 | 4.39 E-03 | Amino Acid | BCAA Metabolism |
| 3-methylglutaconate | 0.60 | 7.67 E-09 | 4.95 E-08 | Amino Acid | BCAA Metabolism |
| 4-methyl-2-oxopentanoate | -0.15 | 9.86 E-03 | 2.08 E-02 | Amino Acid | BCAA Metabolism |
| alpha-hydroxyisovalerate | 0.27 | 4.05 E-04 | 1.12 E-03 | Amino Acid | BCAA Metabolism |
| beta-hydroxyisovalerate | 0.37 | 3.02 E-08 | 1.69 E-07 | Amino Acid | BCAA Metabolism |
| Ethylmalonate | 0.44 | 1.48 E-15 | 3.44 E-14 | Amino Acid | BCAA Metabolism |
| isobutyrylglycine (C4) | 0.44 | 2.39 E-07 | 1.11 E-06 | Amino Acid | BCAA Metabolism |
| Isovalerylglycine | 0.47 | 1.90 E-05 | 6.52 E-05 | Amino Acid | BCAA Metabolism |
| Methylmalonate | 0.33 | 1.70 E-06 | 7.04 E-06 | Lipid | BCAA Metabolism |
| Methylsuccinate | 0.32 | 6.09 E-04 | 1.64 E-03 | Amino Acid | BCAA Metabolism |
| N-acetylisoleucine | 0.55 | 1.05 E-16 | 3.47 E-15 | Amino Acid | BCAA Metabolism |
| N-acetylleucine | 0.67 | 6.17 E-13 | 8.46 E-12 | Amino Acid | BCAA Metabolism |
| N-acetylvaline | 0.67 | 2.98 E-23 | 2.54 E-21 | Amino Acid | BCAA Metabolism |
| propionylglycine (C3) | 0.20 | 2.52 E-03 | 6.10 E-03 | Lipid | BCAA Metabolism |
| Kynurenate | 0.77 | 3.23 E-07 | 1.49 E-06 | Amino Acid | Kynurenine Metabolism |
| Kynurenine | 0.44 | 6.86 E-08 | 3.56 E-07 | Amino Acid | Kynurenine Metabolism |
| N-acetylkynurenine | 0.75 | 2.17 E-08 | 1.27 E-07 | Amino Acid | Kynurenine Metabolism |
| acetylcarnitine (C2) | 0.34 | 1.64 E-07 | 7.90 E-07 | Lipid | Short-chain Acylcarnitine |
| propionylcarnitine (C3) | 0.29 | 2.01 E-06 | 8.25 E-06 | Lipid | Short-chain Acylcarnitine |
| 3-hydroxybutyrylcarnitine (C3-DC) | 0.52 | 6.81 E-09 | 4.55 E-08 | Lipid | Short-chain Acylcarnitine |
| 3-hydroxybutyrylcarnitine (C3-DC) | 0.48 | 9.79 E-05 | 3.00 E-04 | Lipid | Short-chain Acylcarnitine |
| malonylcarnitine (C3-DC) | 0.42 | 2.27 E-16 | 6.23 E-15 | Lipid | Short-chain Acylcarnitine |
| butyrylcarnitine (C4) | 0.32 | 2.51 E-06 | 1.00 E-05 | Lipid | Short-chain Acylcarnitine |
| isobutyrylcarnitine (C4) | 0.39 | 5.45 E-07 | 2.41 E-06 | Amino Acid | Short-chain Acylcarnitine |
| 2-methylmalonylcarnitine (C4-DC) | 0.63 | 1.74 E-15 | 3.70 E-14 | Lipid | Short-chain Acylcarnitine |
| succinylcarnitine (C4-DC) | 0.41 | 1.07 E-09 | 8.64 E-09 | Energy | Short-chain Acylcarnitine |
| 2-methylbutyroylcarnitine (C5) | 0.52 | 3.96 E-12 | 4.99 E-11 | Amino Acid | Short-chain Acylcarnitine |
| glutaroylcarnitine (C5) | 0.46 | 6.10 E-12 | 7.43 E-11 | Amino Acid | Short-chain Acylcarnitine |
| isovalerylcarnitine (C5) | 0.33 | 5.04 E-06 | 1.91 E-05 | Amino Acid | Short-chain Acylcarnitine |
| tiglyl carnitine (C5) | 0.41 | 1.51 E-09 | 1.18 E-08 | Amino Acid | Short-chain Acylcarnitine |
| 3-methylglutarylcarnitine (C6-DC) | 0.64 | 2.73 E-11 | 3.08 E-10 | Amino Acid | Short-chain Acylcarnitine |
| hexanoylcarnitine (C6) | 0.50 | 6.99 E-11 | 7.36 E-10 | Lipid | Short-chain Acylcarnitine |
| Arabinose | 0.48 | 1.60 E-13 | 2.40 E-12 | Carbohydrate | Pentose Metabolism |
| arabitol/xylitol | 0.56 | 1.62 E-16 | 4.77 E-15 | Carbohydrate | Pentose Metabolism |
| arabonate/xylonate | 0.66 | 4.27 E-18 | 1.56 E-16 | Carbohydrate | Pentose Metabolism |
| erythronate* | 0.60 | 2.12 E-19 | 9.57 E-18 | Carbohydrate | Pentose Metabolism |
| ribitol | 0.38 | 3.20 E-10 | 2.79 E-09 | Carbohydrate | Pentose Metabolism |
| ribonate (ribonolactone) | 0.57 | 3.69 E-18 | 1.42 E-16 | Carbohydrate | Pentose Metabolism |
| ribulonate/xylulonate* | 0.38 | 2.39 E-08 | 1.38 E-07 | Carbohydrate | Pentose Metabolism |
| sedoheptulose | 0.23 | 1.43 E-03 | 3.60 E-03 | Carbohydrate | Pentose Metabolism |
| xylose | 0.29 | 5.32 E-05 | 1.70 E-04 | Carbohydrate | Pentose Metabolism |

**Additional file 7. Highlighted Significantly Different Metabolites with increased N-formylmethionine abundance in VITdAL-ICU Cohort at day 0 (Continued)**

| **Metabolite** | **Beta Coefficient** | **p-value** | **q-value** | **Super Pathway** | **Sub Pathway** |
| --- | --- | --- | --- | --- | --- |
| 1-methyladenosine | 0.43 | 1.86 E-16 | 5.30 E-15 | Nucleotide | Purine Metabolism |
| 7-methylguanine | 0.39 | 5.22 E-08 | 2.82 E-07 | Nucleotide | Purine Metabolism |
| Adenine | 0.41 | 6.34 E-09 | 4.31 E-08 | Nucleotide | Purine Metabolism |
| Adenosine | 0.39 | 1.23 E-08 | 7.59 E-08 | Nucleotide | Purine Metabolism |
| Allantoin | 0.27 | 1.14 E-08 | 7.06 E-08 | Nucleotide | Purine Metabolism |
| Hypoxanthine | 0.25 | 3.80 E-05 | 1.26 E-04 | Nucleotide | Purine Metabolism |
| Inosine | 0.15 | 1.49 E-02 | 3.07 E-02 | Nucleotide | Purine Metabolism |
| N1-methylinosine | 0.74 | 3.50 E-18 | 1.41 E-16 | Nucleotide | Purine Metabolism |
| N2,N2-dimethylguanosine | 0.73 | 4.56 E-15 | 9.22 E-14 | Nucleotide | Purine Metabolism |
| N6-carbamoylthreonyladenosine | 0.78 | 1.37 E-24 | 1.32 E-22 | Nucleotide | Purine Metabolism |
| N6-succinyladenosine | 0.60 | 8.53 E-10 | 7.04 E-09 | Nucleotide | Purine Metabolism |
| urate | 0.15 | 3.10 E-03 | 7.42 E-03 | Nucleotide | Purine Metabolism |
| xanthine | 0.34 | 1.46 E-03 | 3.66 E-03 | Nucleotide | Purine Metabolism |
| xanthosine | 0.57 | 3.36 E-07 | 1.54 E-06 | Nucleotide | Purine Metabolism |
| 1-arachidonoyl-GPC* (20:4)* | -0.25 | 2.11 E-04 | 6.11 E-04 | Lipid | Lysophosphatidylcholine |
| 1-lignoceroyl-GPC (24:0) | -0.20 | 5.55 E-03 | 1.22 E-02 | Lipid | Lysophosphatidylcholine |
| 1-palmitoyl-GPC (16:0) | -0.18 | 1.94 E-03 | 4.76 E-03 | Lipid | Lysophosphatidylcholine |
| 1-stearoyl-GPC (18:0) | -0.21 | 1.25 E-03 | 3.20 E-03 | Lipid | Lysophosphatidylcholine |
| 2-palmitoyl-GPC* (16:0)* | -0.20 | 9.86 E-04 | 2.58 E-03 | Lipid | Lysophosphatidylcholine |

Note: Significant associations between N-formylmethionine abundance and the 983 individual metabolites at day 0 in the VITdAL-ICU cohort were determined utilizing linear regression correcting for age, sex, baseline 25(OH)D, SAPS II, and admission diagnosis. A false discovery rate adjusted p-value (q-value) threshold of 0.05 was used to identify all significant differences. For the Acylcarnitine sub pathway: a capital C is followed by the number of carbons within the fatty acyl group attached to the carnitine. DC following the carbon number is a dicarboxylic acylcarnitine. Otherwise for lipids (e.g., d18:1) the letter 'd' refers to the 2 (di-) hydroxyl groups, the number '18' represents the number of carbon atoms and the number '1' indicates the number of double bonds. GPC is glycerophosphocholine. * indicates metabolites are identified via predictive or externally acquired structure evidence when a reference standard does not exist.
