# Supplementary material for: Circulating N-formylmethionine and metabolic shift in critical illness: a multicohort metabolomics study
Source: Crit Care. 2022 Oct 19;26:321. doi: 10.1186/s13054-022-04174-y (PMC9580206; doi:10.1186/s13054-022-04174-y)
Supplement: Supplementary file 9 — Additional file 9. Metabolites significantly changed with increased N-formylmethionine abundance in the VITdAL-ICU Cohort over days 0, 3 and 7. [file 13054_2022_4174_MOESM9_ESM.docx]

**Additional file 9. Metabolites significantly changed with increased N-formylmethionine abundance in the VITdAL-ICU Cohort over days 0, 3 and 7**

| **Metabolite** | **Beta Coefficient** | **p-value** | **q-value** | **Super Pathway** | **Sub Pathway** |
| --- | --- | --- | --- | --- | --- |
| 2,3-dihydroxy-2-methylbutyrate | 0.40 | 7.12 E-26 | 3.99 E-25 | Amino Acid | BCAA Metabolism |
| 2-hydroxy-3-methylvalerate | 0.35 | 2.09 E-26 | 1.21 E-25 | Amino Acid | BCAA Metabolism |
| 3-hydroxy-2-ethylpropionate | 0.31 | 1.49 E-25 | 8.28 E-25 | Amino Acid | BCAA Metabolism |
| 3-hydroxyisobutyrate | 0.31 | 1.33 E-19 | 5.98 E-19 | Amino Acid | BCAA Metabolism |
| 3-methylglutaconate | 0.55 | 8.49 E-68 | 1.89 E-66 | Amino Acid | BCAA Metabolism |
| alpha-hydroxyisovalerate | 0.26 | 1.98 E-16 | 7.76 E-16 | Amino Acid | BCAA Metabolism |
| beta-hydroxyisovalerate | 0.41 | 2.04 E-43 | 1.92 E-42 | Amino Acid | BCAA Metabolism |
| ethylmalonate | 0.40 | 1.72 E-40 | 1.47 E-39 | Amino Acid | BCAA Metabolism |
| isobutyrylglycine (C4) | 0.51 | 1.67 E-37 | 1.30 E-36 | Amino Acid | BCAA Metabolism |
| isovalerate (C5) | 0.12 | 3.88 E-03 | 6.78 E-03 | Amino Acid | BCAA Metabolism |
| isovalerylglycine | 0.51 | 5.56 E-32 | 3.73 E-31 | Amino Acid | BCAA Metabolism |
| methylmalonate | 0.35 | 2.39 E-18 | 1.02 E-17 | Lipid | BCAA Metabolism |
| methylsuccinate | 0.27 | 5.50 E-13 | 1.90 E-12 | Amino Acid | BCAA Metabolism |
| N-acetylisoleucine | 0.62 | 2.51 E-139 | 2.23 E-137 | Amino Acid | BCAA Metabolism |
| N-acetylleucine | 0.71 | 2.92 E-165 | 3.18 E-163 | Amino Acid | BCAA Metabolism |
| N-acetylvaline | 0.71 | 2.51 E-282 | 2.46 E-279 | Amino Acid | BCAA Metabolism |
| propionylglycine (C3) | 0.22 | 5.78 E-09 | 1.62 E-08 | Lipid | BCAA Metabolism |
| valine | 0.06 | 1.03 E-02 | 1.71 E-02 | Amino Acid | BCAA Metabolism |
| kynurenate | 0.66 | 4.11 E-72 | 1.06 E-70 | Amino Acid | Kynurenine Pathway |
| kynurenine | 0.41 | 1.44 E-53 | 1.93 E-52 | Amino Acid | Kynurenine Pathway |
| N-acetylkynurenine | 0.78 | 1.89 E-62 | 3.37 E-61 | Amino Acid | Kynurenine Pathway |
| N-formylanthranilic acid | 0.47 | 4.42 E-43 | 4.13 E-42 | Amino Acid | Kynurenine Pathway |
| acetylcarnitine (C2) | 0.34 | 4.77 E-26 | 2.70 E-25 | Lipid | Short-chain Acylcarnitine |
| 3-hydroxybutyrylcarnitine (C3-DC) | 0.47 | 1.41 E-37 | 1.11 E-36 | Lipid | Short-chain Acylcarnitine |
| malonylcarnitine (C3-DC) | 0.43 | 1.38 E-41 | 1.24 E-40 | Lipid | Short-chain Acylcarnitine |
| propionylcarnitine (C3) | 0.36 | 1.62 E-23 | 8.25 E-23 | Lipid | Short-chain Acylcarnitine |
| isobutyrylcarnitine (C4) | 0.46 | 5.21 E-26 | 2.94 E-25 | Amino Acid | Short-chain Acylcarnitine |
| 2-methylmalonylcarnitine (C4-DC) | 0.67 | 2.14 E-75 | 6.00 E-74 | Lipid | Short-chain Acylcarnitine |
| succinylcarnitine (C4-DC) | 0.41 | 1.45 E-36 | 1.07 E-35 | Energy | Short-chain Acylcarnitine |
| 3-hydroxybutyrylcarnitine (C4-OH) | 0.38 | 3.24 E-13 | 1.14 E-12 | Lipid | Short-chain Acylcarnitine |
| isovalerylcarnitine (C5) | 0.45 | 1.95 E-34 | 1.38 E-33 | Amino Acid | Short-chain Acylcarnitine |
| 2-methylbutyroylcarnitine (C5) | 0.62 | 6.95 E-66 | 1.45 E-64 | Amino Acid | Short-chain Acylcarnitine |
| tiglyl carnitine (C5) | 0.52 | 4.54 E-53 | 5.93 E-52 | Amino Acid | Short-chain Acylcarnitine |
| glutaroylcarnitine (C5) | 0.50 | 1.19 E-58 | 1.82 E-57 | Amino Acid | Short-chain Acylcarnitine |
| 3-methylglutarylcarnitine (C6-DC) | 0.60 | 9.52 E-58 | 1.44 E-56 | Amino Acid | Short-chain Acylcarnitine |
| adipoylcarnitine (C6-DC) | 0.57 | 4.53 E-52 | 5.62 E-51 | Lipid | Short-chain Acylcarnitine |
| hexanoylcarnitine (C6) | 0.47 | 3.41 E-44 | 3.31 E-43 | Lipid | Short-chain Acylcarnitine |
| 3-methyladipoylcarnitine (C7-DC) | 0.61 | 2.62 E-67 | 5.70 E-66 | Lipid | Short-chain Acylcarnitine |
| arabinose | 0.37 | 5.56 E-22 | 7.84 E-21 | Carbohydrate | Pentose Pathway |
| arabitol/xylitol | 0.52 | 1.72 E-31 | 4.52 E-29 | Carbohydrate | Pentose Pathway |
| arabonate/xylonate | 0.58 | 7.65 E-38 | 2.27 E-35 | Carbohydrate | Pentose Pathway |
| ribitol | 0.37 | 7.26 E-19 | 8.05 E-18 | Carbohydrate | Pentose Pathway |
| ribonate | 0.49 | 1.07 E-38 | 6.39 E-37 | Carbohydrate | Pentose Pathway |
| ribulonate/xylulonate* | 0.36 | 2.61 E-15 | 2.57 E-14 | Carbohydrate | Pentose Pathway |
| Sedoheptulose | 0.23 | 4.24 E-06 | 1.50 E-05 | Carbohydrate | Pentose Pathway |
| Xylose | 0.24 | 1.18 E-09 | 5.84 E-09 | Carbohydrate | Pentose Pathway |
| 1-methyladenosine | 0.41 | 1.44 E-108 | 8.80 E-107 | Nucleotide | Purine Metabolism |
| 7-methylguanine | 0.30 | 2.96 E-34 | 2.06 E-33 | Nucleotide | Purine Metabolism |
| Allantoin | 0.28 | 3.93 E-23 | 1.98 E-22 | Nucleotide | Purine Metabolism |
| N1-methylinosine | 0.66 | 1.61 E-126 | 1.31 E-124 | Nucleotide | Purine Metabolism |
| N2,N2-dimethylguanosine | 0.55 | 1.03 E-84 | 3.61 E-83 | Nucleotide | Purine Metabolism |
| N6-carbamoylthreonyladenosine | 0.77 | 3.22 E-190 | 7.90 E-188 | Nucleotide | Purine Metabolism |
| N6-succinyladenosine | 0.60 | 1.51 E-68 | 3.43 E-67 | Nucleotide | Purine Metabolism |

**Additional file 9. Metabolites significantly changed with increased N-formylmethionine abundance in the VITdAL-ICU Cohort over days 0, 3 and 7 (Continued)**

| **Metabolite** | **Beta Coefficient** | **p-value** | **q-value** | **Super Pathway** | **Sub Pathway** |
| --- | --- | --- | --- | --- | --- |
| 1-palmitoyl-GPC (16:0) | -0.16 | 2.59 E-11 | 8.35 E-11 | Lipid | Lysophosphatidylcholine |
| 2-palmitoyl-GPC (16:0)* | -0.22 | 3.21 E-12 | 1.09 E-11 | Lipid | Lysophosphatidylcholine |
| 1-palmitoleoyl-GPC (16:1)* | -0.17 | 2.98 E-08 | 7.92 E-08 | Lipid | Lysophosphatidylcholine |
| 1-stearoyl-GPC (18:0) | -0.16 | 8.88 E-10 | 2.69 E-09 | Lipid | Lysophosphatidylcholine |
| 1-linoleoyl-GPC (18:2) | -0.08 | 3.89 E-03 | 6.78 E-03 | Lipid | Lysophosphatidylcholine |
| 1-linolenoyl-GPC (18:3)* | -0.10 | 5.64 E-03 | 9.60 E-03 | Lipid | Lysophosphatidylcholine |
| 1-arachidonoyl-GPC (20:4)* | -0.16 | 1.01 E-07 | 2.60 E-07 | Lipid | Lysophosphatidylcholine |
| 1-lignoceroyl-GPC (24:0) | -0.13 | 4.11 E-04 | 8.06 E-04 | Lipid | Lysophosphatidylcholine |

Note: Significant associations presented between N-formylmethionine abundance and the 983 individual metabolites determined by mixed effects modeling in the VITdAL-ICU cohort (N=428). For day 0, 3 and 7 repeated measures data, correlations between N-formylmethionine abundance and individual metabolites were determined utilizing linear mixed effects models correcting for age, sex, baseline 25(OH)D, absolute increase in 25(OH)D at day 3, SAPS II, plasma day, admission diagnosis, and an individual subject-specific random-intercept. A false discovery rate adjusted p-value (q-value) threshold of 0.05 was used to identify all significant differences. For the Acylcarnitine sub pathway: a capital C is followed by the number of carbons within the fatty acyl group attached to the carnitine. DC following the carbon number is a dicarboxylic acylcarnitine and an OH following the carbon number indicates a hydroxyl group. Otherwise for lipids (e.g., d18:1) the letter 'd' refers to the 2 (di-) hydroxyl groups, the number '18' represents the number of carbon atoms and the number '1' indicates the number of double bonds. GPC is glycerophosphocholine. * indicates metabolites are identified via predictive or externally acquired structure evidence when a reference standard does not exist.
