# Supplementary material for: Circulating N-formylmethionine and metabolic shift in critical illness: a multicohort metabolomics study
Source: Crit Care. 2022 Oct 19;26:321. doi: 10.1186/s13054-022-04174-y (PMC9580206; doi:10.1186/s13054-022-04174-y)
Supplement: Supplementary file 11 — Additional file 11. Day 0 N-formylmethionine-specific Metabolic Networks with similar effects via Gaussian graphical models. [file 13054_2022_4174_MOESM11_ESM.docx]

**Additional file 11. Day 0 N-formylmethionine-specific Metabolic Networks with similar effects via Gaussian graphical models**

| **Module** | **Module q-value** | **Metabolite** | **Super Pathway** | **Sub-pathway** | **Component β Coefficient** | **Component q-value** |
| --- | --- | --- | --- | --- | --- | --- |
| **A** | 1.20 E-05 | Isovalerylglycine | Amino Acid | BCAA Metabolism | 0.46 | 3.50 E-09 |
|  |  | isobutyrylglycine (C4) | Amino Acid | BCAA Metabolism | 0.43 | 1.68 E-10 |
|  |  | isobutyrylcarnitine (C4) | Amino Acid | Short-Chain Acylcarnitine | 0.39 | 2.12 E-08 |
|  |  |  |  |  |  |  |
| **B** | 1.10 E-21 | N-acetylputrescine | Amino Acid | Polyamine Metabolism | 0.26 | 1.77 E-04 |
|  |  | N-acetyl-beta-alanine | Nucleotide | Pyrimidine Metabolism | 0.41 | 2.23 E-14 |
|  |  | 4-acetamidobutanoate | Amino Acid | Polyamine Metabolism | 0.61 | 2.15 E-22 |
|  |  | (N(1) + N(8))-acetylspermidine | Amino Acid | Polyamine Metabolism | 0.49 | 1.38 E-13 |
|  |  | Acisoga | Amino Acid | Polyamine Metabolism | 0.53 | 5.33 E-15 |
|  |  | N-acetyl-isoputreanine* | Amino Acid | Polyamine Metabolism | 0.57 | 1.90 E-19 |
|  |  |  |  |  |  |  |
| **C** | 1.60 E-20 | N-acetylkynurenine | Amino Acid | Kynurenine Pathway | 0.75 | 1.34 E-17 |
|  |  | Kynurenine | Amino Acid | Kynurenine Pathway | 0.43 | 6.70 E-17 |
|  |  | N-formylanthranilic acid | Amino Acid | Kynurenine Pathway | 0.47 | 2.46 E-13 |
|  |  | N-acetyltryptophan | Amino Acid | Tryptophan Metabolism | 1.29 | 9.62 E-07 |
|  |  | Quinolinate | Cofactor | Nicotinate and Nicotinamide Metabolism | 0.92 | 4.22 E-34 |
|  |  | Kynurenate | Amino Acid | Kynurenine Pathway | 0.76 | 5.64 E-19 |
|  |  |  |  |  |  |  |
| **D** | 1.20 E-10 | arabonate/xylonate | Carbohydrate | Pentose Pathway | 0.65 | 1.62 E-33 |
|  |  | Ribonate | Carbohydrate | Pentose Pathway | 0.57 | 1.14 E-31 |
|  |  |  |  |  |  |  |
| **E** | 2.20 E-18 | Sedoheptulose | Carbohydrate | Pentose Pathway | 0.23 | 7.52 E-04 |
|  |  | Erythritol | Carbohydrate | Pentose Pathway | 0.59 | 1.93 E-28 |
|  |  |  |  |  |  |  |
| **F** | 4.90 E-07 | succinylcarnitine (C4) | Energy | Short-Chain Acylcarnitine | 0.41 | 1.39 E-14 |
|  |  | glutaroylcarnitine (C5) | Amino Acid | Short-Chain Acylcarnitine | 0.45 | 1.30 E-16 |
|  |  |  |  |  |  |  |
| **G** | 1.30 E-07 | hexadecanedioate (C16) | Lipid | Fatty Acid, Dicarboxylate | 0.24 | 1.41 E-02 |
|  |  | hexadecenedioate (C16:1-DC)* | Lipid | Fatty Acid, Dicarboxylate | 0.27 | 5.13 E-03 |
|  |  | octadecadienedioate (C18:2-DC)* | Lipid | Fatty Acid, Dicarboxylate | 0.09 | 8.86 E-02 |
|  |  |  |  |  |  |  |
| **H** | 2.00 E-06 | Phenylacetylmethionine | Peptide | Acetylated Peptides | 0.55 | 1.45 E-11 |
|  |  | Phenylacetylglutamine | Peptide | Acetylated Peptides | 0.61 | 3.00 E-20 |
|  |  | methionine sulfoxide | Amino Acid | Methionine Metabolism | 0.29 | 2.05 E-07 |

Note: Module q-value is the FDR adjusted p-value of the GGM module; Metabolite is the name of the metabolite in module; Super Pathway is the name of the major biochemical pathway in the module; Sub-pathway is a subset of the major biochemical pathway in the module; Component q-value and β coefficient results presented following linear regression modeling of each of the 983 individual metabolites measured at day 0. All estimates adjusted for age, sex, SAPS II, admission diagnosis, and 25(OH)D at day 0. A q-value < 0.05 was used to identify all significant associations. All module individual Component metabolites are significant except for octadecadienedioate. For the Short-chain Acylcarnitine sub pathway: a capital C is followed by the number of carbons within the fatty acyl group attached to the carnitine. Otherwise for the lipids (e.g., 18:2) the number ‘18’ represents the number of carbon atoms and the number ‘2’ indicates the number of double bonds present, DC following the carbon number is a dicarboxylate. * indicates metabolites are identified via predictive or externally acquired structure evidence when a reference standard does not exist.
