# Supplementary material for: Circulating N-formylmethionine and metabolic shift in critical illness: a multicohort metabolomics study
Source: Crit Care. 2022 Oct 19;26:321. doi: 10.1186/s13054-022-04174-y (PMC9580206; doi:10.1186/s13054-022-04174-y)
Supplement: Supplementary file 12 — Additional file 12. Significantly Different Metabolites with increased N-formylmethionine abundance in the RoCI Cohort at day 0. [file 13054_2022_4174_MOESM12_ESM.docx]

**Additional file 12. Significantly Different Metabolites with increased N-formylmethionine abundance in the RoCI Cohort at day 0**

| **Metabolite** | **Beta Coefficient** | **p-value** | **q-value** | **Super Pathway** | **Sub Pathway** |
| --- | --- | --- | --- | --- | --- |
| 3-hydroxyisobutyrate | 0.74 | 2.11 E-04 | **2.54 E-03** | Amino Acid | BCAA Metabolism |
| 3-hydroxy-2-ethylpropionate | 0.70 | 6.93 E-07 | **5.40 E-05** | Amino Acid | BCAA Metabolism |
| 2-hydroxy-3-methylvalerate | 0.64 | 4.83 E-03 | **2.43 E-02** | Amino Acid | BCAA Metabolism |
| alpha-hydroxyisocaproate | 0.63 | 6.15 E-03 | **2.81 E-02** | Amino Acid | BCAA Metabolism |
| alpha-hydroxyisovalerate | 0.57 | 2.37 E-02 | 8.16 E-02 | Amino Acid | BCAA Metabolism |
| beta-hydroxyisovalerate | 0.53 | 7.41 E-03 | **3.26 E-02** | Amino Acid | BCAA Metabolism |
| lactate | 0.50 | 7.24 E-03 | **3.22 E-02** | Carbohydrate | Glycolysis |
| kynurenate | 1.04 | 8.47 E-04 | **6.56 E-03** | Amino Acid | Kynurenine Metabolism |
| kynurenine | 0.63 | 9.26 E-04 | **6.79 E-03** | Amino Acid | Kynurenine Metabolism |
| succinylcarnitine (C4) | 0.52 | 5.31 E-03 | **2.48 E-02** | Energy | Short-chain Acylcarnitine |
| butyrylcarnitine (C4) | 0.33 | 3.07 E-02 | 9.15 E-02 | Lipid | Short-chain Acylcarnitine |
| tiglyl carnitine (C5) | 0.75 | 3.85 E-04 | **3.68 E-03** | Amino Acid | Short-chain Acylcarnitine |
| glutaroyl carnitine (C5) | 0.68 | 1.61 E-03 | **1.07 E-02** | Amino acid | Short-chain Acylcarnitine |
| 2-methylbutyroylcarnitine (C5) | 0.65 | 4.54 E-03 | **2.38 E-02** | Amino Acid | Short-chain Acylcarnitine |
| hydroxyisovaleroyl carnitine (C5-OH) | 0.61 | 9.61 E-04 | **6.79 E-03** | Amino acid | Short-chain Acylcarnitine |
| hexanoylcarnitine (C6) | 0.57 | 1.59 E-02 | 6.08 E-02 | Lipid | Short-chain Acylcarnitine |
| methylglutaroylcarnitine (C6-DC) | 0.70 | 2.49 E-03 | **1.47 E-02** | Amino acid | Short-chain Acylcarnitine |
| octanoylcarnitine (C8) | 0.66 | 5.13 E-03 | **2.48 E-02** | Lipid | Medium-chain Acylcarnitine |
| decanoylcarnitine (C10) | 0.51 | 1.08 E-02 | **4.39 E-02** | Lipid | Medium-chain Acylcarnitine |
| cis-4-decenoylcarnitine (C10:1) | 0.43 | 2.93 E-03 | **1.67 E-02** | Lipid | Medium-chain Acylcarnitine |
| arabitol/xylitol | 1.08 | 8.76 E-03 | **3.65 E-02** | Carbohydrate | Pentose Metabolism |
| arabonate/xylonate | 0.86 | 1.89 E-05 | **3.84 E-04** | Carbohydrate | Pentose Metabolism |
| arabinose | 0.53 | 3.92 E-03 | **2.09 E-02** | Carbohydrate | Pentose Metabolism |
| erythritol | 0.75 | 3.23 E-06 | **1.05 E-04** | Carbohydrate | Pentose Metabolism |
| xylose | 0.44 | 4.64 E-03 | **2.39 E-02** | Carbohydrate | Pentose Metabolism |
| N2,N2-dimethylguanosine | 0.91 | 4.53 E-06 | **1.34 E-04** | Nucleotide | Purine Metabolism |
| allantoin | 0.88 | 3.71 E-04 | **3.66 E-03** | Nucleotide | Purine Metabolism |
| N6-carbamoylthreonyladenosine | 0.67 | 4.27 E-05 | **6.62 E-04** | Nucleotide | Purine Metabolism |
| 7-methylguanine | 0.39 | 8.13 E-03 | **3.52 E-02** | Nucleotide | Purine Metabolism |
| 1-methyladenosine | 0.35 | 5.53 E-04 | **4.86 E-03** | Nucleotide | Purine Metabolism |
| urate | 0.34 | 2.91 E-02 | 8.98 E-02 | Nucleotide | Purine Metabolism |

Note: Significant associations between N-formylmethionine abundance and the 411 individual metabolites at day 0 were determined in the RoCI cohort utilizing linear regression correcting for age, sex, race, and APACHE II. A false discovery rate adjusted p-value (q-value) threshold of 0.05 was used to identify all significant differences shown in bold. Results with a false discovery rate adjusted p-value (q-value) threshold of up to 0.10 are also shown. For the Acylcarnitine sub pathway: a capital C is followed by the number of carbons within the fatty acyl group attached to the carnitine. DC following the carbon number is a dicarboxylic acylcarnitine and an OH following the carbon number indicates a hydroxyl group. Otherwise for lipids (e.g., d18:1) the letter 'd' refers to the 2 (di-) hydroxyl groups, the number '18' represents the number of carbon atoms and the number '1' indicates the number of double bonds. * indicates metabolites are identified via predictive or externally acquired structure evidence when a reference standard does not exist.
