# Supplementary material for: Circulating N-formylmethionine and metabolic shift in critical illness: a multicohort metabolomics study
Source: Crit Care. 2022 Oct 19;26:321. doi: 10.1186/s13054-022-04174-y (PMC9580206; doi:10.1186/s13054-022-04174-y)
Supplement: Supplementary file 13 — Additional file 13. Significantly Different Metabolites with increased N-formylmethionine at day 0 in both VITdAL-ICU and RoCI Cohorts. [file 13054_2022_4174_MOESM13_ESM.docx]

**Additional file 13. Significantly Different Metabolites with increased N-formylmethionine abundance at day 0 in both VITdAL-ICU and RoCI Cohorts**

| **Metabolite** | **VITdAL-ICU Cohort** | | **RoCI Cohort** | | **Super Pathway** | **Sub Pathway** |
| --- | --- | --- | --- | --- | --- | --- |
|  | **Beta Coefficient** | **q-value** | **Beta Coefficient** | **q-value** |  |  |
| phenylacetylglutamine | 0.61 | **1.30 E-11** | 0.56 | 8.23 E-02 | Peptide | Acetylated Peptides |
| N-acetylalanine | 0.64 | **9.80 E-34** | 0.68 | **1.80 E-09** | Amino Acid | Alanine and Aspartate Metabolism |
| N-acetylaspartate | 0.23 | **3.18 E-04** | 0.48 | 5.58 E-02 | Amino Acid | Alanine and Aspartate Metabolism |
| glucuronate | 0.54 | **5.30 E-10** | 0.56 | **3.22 E-02** | Carbohydrate | Aminosugar Metabolism |
| N-acetylneuraminate | 0.59 | **2.00 E-12** | 0.83 | **1.34 E-04** | Carbohydrate | Aminosugar Metabolism |
| 2-hydroxy-3-methylvalerate | 0.40 | **3.20 E-05** | 0.64 | **2.43 E-02** | Amino Acid | BCAA Metabolism |
| 3-hydroxy-2-ethylpropionate | 0.33 | **7.40 E-07** | 0.70 | **5.40 E-05** | Amino Acid | BCAA Metabolism |
| 3-hydroxyisobutyrate | 0.27 | **4.39 E-03** | 0.74 | **2.54 E-03** | Amino Acid | BCAA Metabolism |
| alpha-hydroxyisovalerate | 0.27 | **1.12 E-03** | 0.57 | 8.16 E-02 | Amino Acid | BCAA Metabolism |
| beta-hydroxyisovalerate | 0.37 | **1.70 E-07** | 0.53 | **3.26 E-02** | Amino Acid | BCAA Metabolism |
| deoxycarnitine | 0.38 | **1.50 E-09** | 0.46 | **2.48 E-02** | Lipid | Carnitine Metabolism |
| creatinine | 0.47 | **1.40 E-13** | 0.61 | **6.79 E-03** | Amino Acid | Creatine Metabolism |
| 2-hydroxyglutarate | 0.13 | 8.96 E-02 | 0.55 | 3.65 E-02 | Lipid | Fatty Acid, Dicarboxylate |
| 3-hydroxydecanoate | 0.19 | 9.53 E-02 | 0.58 | 2.00 E-02 | Lipid | Fatty Acid, Monohydroxy |
| mannitol/sorbitol | 0.33 | **3.56 E-03** | 0.92 | 7.69 E-02 | Carbohydrate | Fructose, Mannose and Galactose Metabolism |
| gamma-glutamylisoleucine* | 0.24 | **5.90 E-05** | 0.63 | **4.80 E-06** | Peptide | Gamma-glutamyl Amino Acid |
| gamma-glutamylleucine | 0.22 | **2.46 E-03** | 0.61 | **5.07 E-03** | Peptide | Gamma-glutamyl Amino Acid |
| gamma-glutamylphenylalanine | 0.39 | **2.20 E-08** | 0.55 | 8.64 E-02 | Peptide | Gamma-glutamyl Amino Acid |
| gamma-glutamyltyrosine | 0.16 | 7.69 E-02 | 0.59 | 6.08 E-02 | Peptide | Gamma-glutamyl Amino Acid |
| gamma-glutamylvaline | 0.20 | **3.23 E-04** | 0.54 | **5.65 E-03** | Peptide | Gamma-glutamyl Amino Acid |
| pyroglutamine* | 0.17 | **7.07 E-03** | 0.58 | **2.47 E-02** | Amino Acid | Glutamate Metabolism |
| 2-hydroxybutyrate /2-hydroxyisobutyrate | 0.14 | 8.60 E-02 | 0.57 | 7.32 E-03 | Amino Acid | Glutathione Metabolism |
| N-acetylserine | 0.71 | **2.20 E-27** | 0.74 | **7.30 E-05** | Amino Acid | Glycine, Serine and Threonine Metabolism |
| N-acetylthreonine | 0.68 | **8.00 E-25** | 0.62 | **3.11 E-03** | Amino Acid | Glycine, Serine and Threonine Metabolism |
| lactate | 0.11 | 5.46 E-02 | 0.50 | **3.22 E-02** | Carbohydrate | Glycolysis |
| hydantoin-5-propionic acid | 0.70 | **3.60 E-08** | 0.77 | **1.29 E-03** | Amino Acid | Histidine Metabolism |
| trans-urocanate | 0.42 | **3.47 E-04** | 1.03 | **4.97 E-03** | Amino Acid | Histidine Metabolism |
| myo-inositol | 0.42 | **1.10 E-05** | 0.98 | **2.75 E-03** | Lipid | Inositol Metabolism |
| kynurenine | 0.44 | **3.60 E-07** | 0.63 | **6.79 E-03** | Amino Acid | Kynurenine Metabolism |
| kynurenate | 0.77 | **1.50 E-06** | 1.04 | **6.56 E-03** | Amino Acid | Kynurenine Metabolism |
| N6,N6,N6-trimethyllysine | 0.49 | **9.20 E-11** | 0.72 | **1.57 E-04** | Amino Acid | Lysine Metabolism |
| 1-arachidonoyl-GPC* (20:4)* | -0.25 | **6.11 E-04** | -0.51 | 7.34 E-02 | Lipid | Lysophosphatidylcholine |
| 1-linoleoyl-GPC (18:2) | -0.12 | 8.42 E-02 | -0.52 | 8.16 E-02 | Lipid | Lysophosphatidylcholine |
| 1-arachidonoyl-GPE (20:4n6)* | -0.11 | **3.60 E-02** | -0.43 | 6.45 E-02 | Lipid | Lysophospholipid |
| succinylcarnitine (C4) | 0.41 | **8.60 E-09** | 0.52 | **2.48 E-02** | Energy | Short-chain Acylcarnitine |
| butyrylcarnitine (C4) | 0.32 | **1.00 E-05** | 0.33 | 9.15 E-02 | Lipid | Short-chain Acylcarnitine |
| tiglyl carnitine (C5) | 0.41 | **1.20 E-08** | 0.75 | **3.68 E-03** | Amino Acid | Short-chain Acylcarnitine |
| 2-methylbutyroylcarnitine (C5) | 0.52 | **5.00 E-11** | 0.65 | **2.38 E-02** | Amino Acid | Short-chain Acylcarnitine |
| hexanoylcarnitine (C6) | 0.50 | **7.40 E-10** | 0.57 | 6.08 E-02 | Lipid | Short-chain Acylcarnitine |
| octanoylcarnitine (C8) | 0.54 | **4.60 E-12** | 0.66 | **2.48 E-02** | Lipid | Medium-chain Acylcarnitine |
| cis-4-decenoylcarnitine (C10:1) | 0.51 | **3.70 E-14** | 0.43 | **1.67 E-02** | Lipid | Medium-chain Acylcarnitine |
| decanoylcarnitine (C10) | 0.52 | **6.10 E-12** | 0.51 | **4.39 E-02** | Lipid | Medium-chain Acylcarnitine |
| cysteine | 0.31 | **5.00 E-06** | 0.89 | **8.70 E-05** | Amino Acid | Methionine, Cysteine, SAM and Taurine Metabolism |
| N-acetylmethionine | 0.97 | **1.60 E-36** | 0.77 | **1.10 E-05** | Amino Acid | Methionine, Cysteine, SAM and Taurine Metabolism |
| N1-Methyl-2-pyridon E-5-carboxamide | 0.46 | **5.40 E-10** | 0.41 | 6.08 E-02 | Cofactors and Vitamins | Nicotinate and Nicotinamide Metabolism |
| trigonelline (N'-methylnicotinate) | 0.34 | **1.77 E-02** | 0.34 | 9.15 E-02 | Cofactors and Vitamins | Nicotinate and Nicotinamide Metabolism |

**Additional file 14. Significantly Different Metabolites with increased N-formylmethionine abundance at day 0 in both VITdAL-ICU and RoCI Cohorts (Continued)**

| **Metabolite** | **VITdAL-ICU Cohort** | | **RoCI Cohort** | | **Super Pathway** | **Sub Pathway** |
| --- | --- | --- | --- | --- | --- | --- |
|  | **Beta Coefficient** | **q-value** | **Beta Coefficient** | **q-value** |  |  |
| phosphate | 0.10 | 7.77 E-02 | 0.61 | **3.33 E-03** | Energy | Oxidative Phosphorylation |
| arabinose | 0.48 | **2.40 E-12** | 0.53 | **2.09 E-02** | Carbohydrate | Pentose Metabolism |
| arabitol/xylitol | 0.56 | **4.80 E-15** | 1.08 | **3.65 E-02** | Carbohydrate | Pentose Metabolism |
| arabonate/xylonate | 0.66 | **1.60 E-16** | 0.84 | **9.57 E-04** | Carbohydrate | Pentose Metabolism |
| erythritol | 0.60 | **2.53 E-14** | 0.75 | **1.05 E-04** | Carbohydrate | Pentose Metabolism |
| erythronate* | 0.60 | **9.60 E-18** | 0.83 | **6.50 E-05** | Carbohydrate | Pentose Metabolism |
| xylose | 0.29 | **1.70 E-04** | 0.44 | **2.40 E-02** | Carbohydrate | Pentose Metabolism |
| N-acetylphenylalanine | 0.66 | **7.30 E-13** | 0.74 | **6.94 E-03** | Amino Acid | Phenylalanine Metabolism |
| phenyllactate | 0.53 | **5.20 E-08** | 0.88 | **1.10 E-02** | Amino Acid | Phenylalanine Metabolism |
| glycerophosphorylcholine | -0.25 | **2.97 E-04** | -0.51 | **2.59 E-04** | Lipid | Phospholipid Metabolism |
| 4-acetamidobutanoate | 0.61 | **2.30 E-09** | 0.80 | **1.08 E-02** | Amino Acid | Polyamine Metabolism |
| 21-hydroxypregnenolone disulfate | 0.44 | **1.50 E-06** | 0.72 | **3.66 E-02** | Lipid | Pregnenolone Steroids |
| 1-methyladenosine | 0.43 | **5.30 E-15** | 0.35 | **4.86 E-03** | Nucleotide | Purine Metabolism |
| 7-methylguanine | 0.39 | **2.80 E-07** | 0.39 | **3.52 E-02** | Nucleotide | Purine Metabolism |
| allantoin | 0.27 | **7.10 E-08** | 0.88 | **3.66 E-03** | Nucleotide | Purine Metabolism |
| N2,N2-dimethylguanosine | 0.73 | **9.20 E-14** | 0.91 | **1.34 E-04** | Nucleotide | Purine Metabolism |
| N6-carbamoylthreonyladenosine | 0.78 | **1.30 E-22** | 0.67 | **6.62 E-04** | Nucleotide | Purine Metabolism |
| urate | 0.15 | **7.42 E-03** | 0.34 | 8.98 E-02 | Nucleotide | Purine Metabolism |
| 3-ureidopropionate | 0.36 | **2.64 E-03** | 0.51 | 6.20 E-02 | Nucleotide | Pyrimidine Metabolism |
| N-acetyl-beta-alanine | 0.42 | **1.90 E-07** | 0.42 | **1.79 E-02** | Nucleotide | Pyrimidine Metabolism |
| pseudouridine | 0.64 | **6.40 E-25** | 0.69 | **5.60 E-04** | Nucleotide | Pyrimidine Metabolism |
| deoxycholate | -0.31 | **2.90 E-05** | -0.51 | 8.16 E-02 | Lipid | Secondary Bile Acid Metabolism |
| glycocholenate sulfate* | 0.38 | **9.20 E-06** | 0.50 | 8.16 E-02 | Lipid | Secondary Bile Acid Metabolism |
| taurocholenate sulfate* | 0.45 | **6.30 E-05** | 0.62 | 6.65 E-02 | Lipid | Secondary Bile Acid Metabolism |
| cholesterol | 0.19 | **1.64 E-04** | 0.27 | **1.71 E-02** | Lipid | Sterol |
| C-glycosyltryptophan | 0.65 | **5.80 E-19** | 0.68 | **1.29 E-03** | Amino Acid | Tryptophan Metabolism |
| indolelactate | 0.44 | **8.90 E-08** | 0.87 | **2.59 E-04** | Amino Acid | Tryptophan Metabolism |
| N-acetyltyrosine | 0.65 | **5.10 E-11** | 0.84 | **5.40 E-05** | Amino Acid | Tryptophan Metabolism |
| 3-(4-hydroxyphenyl)lactate | 0.54 | **2.70 E-08** | 1.02 | **2.14 E-04** | Amino Acid | Tyrosine Metabolism |
| 3-methoxytyrosine | 0.14 | 5.80 E-02 | 0.39 | 7.75 E-02 | Amino Acid | Tyrosine Metabolism |
| 4-hydroxyphenylpyruvate | 0.44 | **4.70 E-07** | 0.60 | 9.15 E-02 | Amino Acid | Tyrosine Metabolism |
| vanillylmandelate | 0.80 | **5.90 E-08** | 0.75 | **1.08 E-02** | Amino Acid | Tyrosine Metabolism |
| dimethylarginine | 0.34 | **4.10 E-09** | 0.62 | **4.36 E-04** | Amino Acid | Urea cycle; Arginine and Proline Metabolism |
| urea | 0.45 | **2.00 E-12** | 0.78 | **2.54 E-03** | Amino Acid | Urea cycle; Arginine and Proline Metabolism |

Note: For the VITdAL-ICU cohort, significant associations between N-formylmethionine abundance and the 983 individual metabolites at day 0 were determined utilizing linear regression correcting for age, sex, baseline 25(OH)D, SAPS II, and admission diagnosis. For the RoCI cohort, significant associations between N-formylmethionine abundance and the 411 individual metabolites at day 0 were determined utilizing linear regression correcting for age, sex, race, and APACHE II. A false discovery rate adjusted p-value (q-value) threshold of 0.05 was used to identify all significant differences (q-value < 0.05) and is noted in the table with bold text. Results with a false discovery rate adjusted p-value (q-value) threshold of up to 0.10 are also shown. For the Short-chain Acylcarnitine sub pathway: a capital C is followed by the number of carbons within the fatty acyl group attached to the carnitine. GPC is glycerophosphocholine and GPE is glycerophosphoethanolamine. Otherwise for the lipids (e.g., 18:2) the number ‘18’ represents the number of carbon atoms and the number ‘2’ indicates the number of double bonds present. * indicates metabolites are identified via predictive or externally acquired structure evidence when a reference standard does not exist.
