# Supplementary figures and images for: Circulating N-formylmethionine and metabolic shift in critical illness: a multicohort metabolomics study
Source: Crit Care. 2022 Oct 19;26:321. doi: 10.1186/s13054-022-04174-y (PMC9580206; doi:10.1186/s13054-022-04174-y)

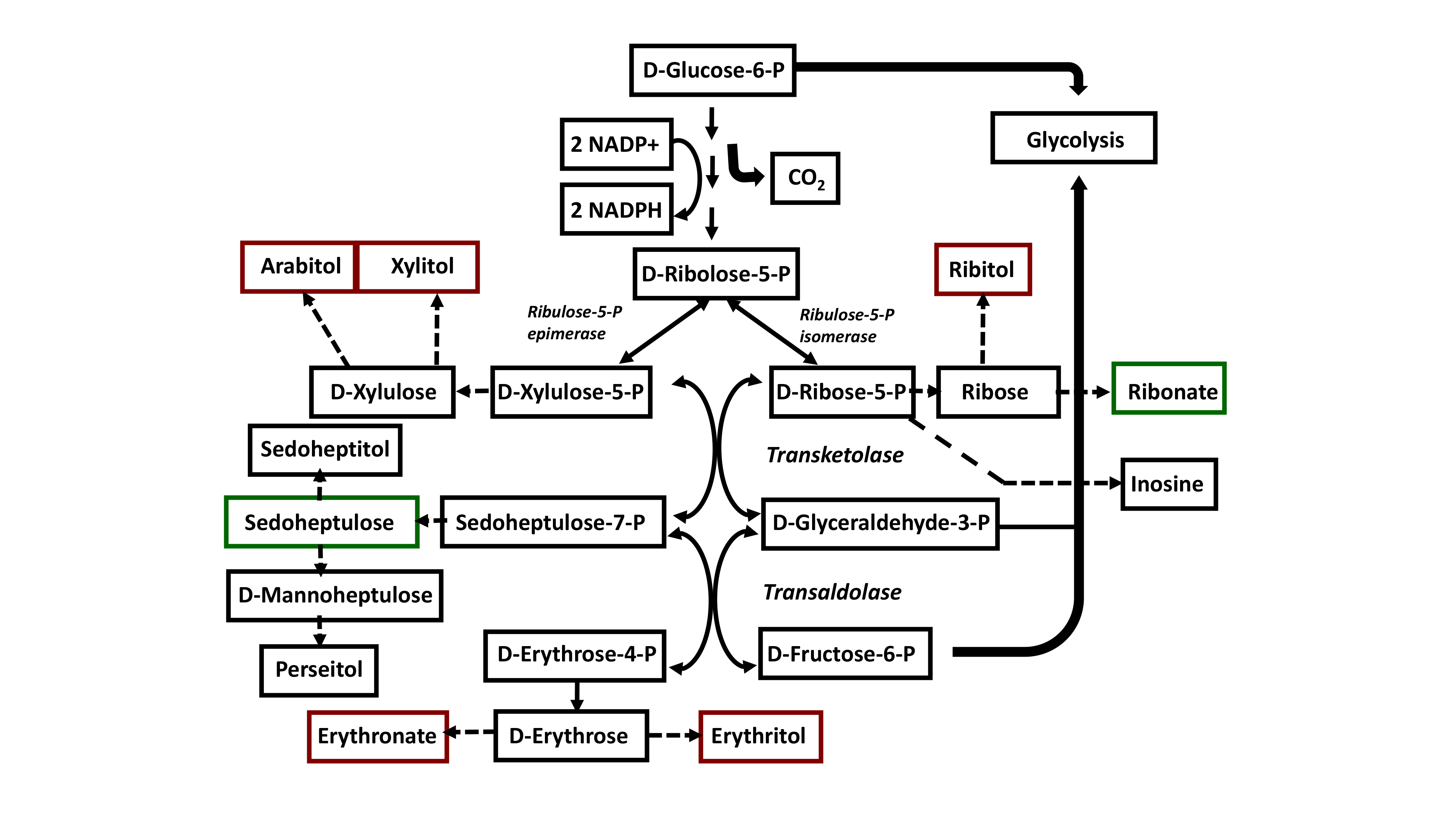

Supplement: Supplementary file 14 — Additional file 14. Increased Pentose Phosphate Pathway Metabolites with N-formylmethionine. Metabolites increased in the VITdAL-ICU and RoCI cohorts with increased N-formylmethionine abundance are highlighted in red, metabolites increased in the VITdAL-ICU cohort with increased N-formylmethionine abundance are highlighted in green. The Pentose Phosphate Pathway produces NADPH and ribose 5-phosphate for redox regulation and biosynthesis, respectively. [file 13054_2022_4174_MOESM14_ESM.tif]
